# Comparison of diffuse correlation spectroscopy analytical models for cerebral blood flow measurements

**DOI:** 10.1117/1.JBO.30.11.115002

**Published:** 2025-11-12

**Authors:** Mingliang Pan, Quan Wang, Yuanzhe Zhang, David Day-Uei Li

**Affiliations:** University of Strathclyde, Department of Biomedical Engineering, Glasgow, United Kingdom

**Keywords:** cerebral blood flow, diffuse correlation spectroscopy, analytical models, Monte Carlo simulation

## Abstract

**Significance:**

Although multi-layer diffuse correlation spectroscopy (DCS) analytical models have been proposed to reduce contamination from superficial signals when probing cerebral blood flow index (CBFi), a comprehensive comparison and clear guidance for model selection remain lacking. This report aims to address this gap.

**Aim:**

We aim to systematically compare three DCS analytical models: the semi-infinite, two-layer, and three-layer models, with a focus on their fundamental differences, data processing approaches, and the accuracy and reliability of CBFi estimation. We also provide practical recommendations for selecting the most appropriate model based on specific application scenarios to support researchers in applying DCS effectively.

**Approach:**

Experimental data were generated by simulating a four-layer slab head model using the Monte Carlo eXtreme toolkit. We evaluated various fitting strategies for three DCS models: early time lag range (ETLR) fitting with or without treating the coherence factor β as a fitting parameter for the semi-infinite model, single-distance (SD) and multi-distance (MD) fitting for the two- and three-layer models. We then compared their performance in terms of CBF sensitivity, recovery of relative CBFi (rCBFi) changes, accuracy of absolute CBFi estimates across different source-to-detector separations (ρ=20, 25, 30, and 35 mm), ability to separate the crosstalk from extracerebral layers [scalp BFi (SBFi), and skull BFi (BBFi)], sensitivity to parameter assumption errors, and time-to-result, using the respective optimal fitting strategies for each model.

**Results:**

The optimal fitting methods for estimating CBFi are ETLR fitting with a constant β for the semi-infinite model, SD fitting with β fixed for the two-layer model, and MD fitting for the three-layer model. The two-layer and three-layer models exhibit enhanced CBFi sensitivity, approaching 100%, compared with 36.8% for the semi-infinite model at ρ=30  mm. The semi-infinite model is suitable only for rCBFi recovery at a larger ρ (≥30  mm). In contrast, the two-layer model is appropriate for both CBFi and rCBFi recovery across all tested ρ values (20, 25, 30, and 35 mm in this work), although its robustness declines as ρ increases. The three-layer model enables simultaneous recovering of CBFi, SBFi, and rCBFi. Among these, the two-layer model is the most effective at mitigating the influence of extracerebral BFi, whereas CBFi estimates from the semi-infinite and three-layer models remain consistently affected by variations in SBFi and BBFi. Errors in assumed model parameters have minimal impact on rCBFi recovery across all models. In terms of computational efficiency, the semi-infinite model requires only 0.38 s of processing 500 data samples, demonstrating potential for real-time rCBFi inference. In comparison, the two-layer and three-layer models require substantially longer processing times of 9502.18 and 35,099.34 s, respectively.

**Conclusions:**

This systematic comparison of three DCS analytical models demonstrates the superior ability of multi-layer models to reduce the influence of superficial tissue layers, thereby enhancing CBFi and rCBFi sensitivity relative to the semi-infinite model. We evaluated various fitting strategies, and beyond recommending the optimal approach for each model, we provide practical guidance for selecting the most appropriate model based on specific objectives, experimental conditions, and data analysis requirements. We believe we offer a valuable reference for researchers in the field, supporting informed model selection and highlighting key considerations for the effective application of DCS analytical models.

## 1 Introduction

Blood flow (BF) is a critical clinical biomarker of human health, responsible for transporting oxygen, nutrients, and metabolic byproducts through the bloodstream.^1^ Diffuse correlation spectroscopy (DCS) is a non-invasive optical technique that uses near-infrared light to assess deep tissue BF.^2^ It quantifies BF by calculating the normalized intensity autocorrelation function (ACF, $g_{2}$) of fluctuations in scattered coherent light, primarily arising from the motion of red blood cells^3^ and offers a high temporal resolution ranging from 1 to 100 Hz.^4^^–^^6^ DCS has been broadly applied to monitor brain health/functions,^7^^–^^9^ cardio-cerebrovascular diseases,^10^^–^^13^ neurovascular coupling,^14^^,^^15^ and tumor diagnosis^16^ and therapy.^17^

Traditionally, the measured $g_{2}$ is fitted to an analytical solution assuming a semi-infinite homogeneous tissue model to extract the BF index (BFi).^18^^,^^19^ However, this simplified model neglects the anatomical complexity of human tissue. For example, it is insufficient for analyzing cerebral BFi (CBFi), as the human head comprises multiple layers, including the scalp, skull, cerebrospinal fluid, grey matter, and white matter.^20^^,^^21^ Due to the tissue heterogeneity, DCS signals originating from deeper brain layers can be contaminated by the BF in superficial layers (i.e., scalp and skull),^22^^–^^28^ often leading to underestimation of CBFi.^24^^,^^25^^,^^29^

Various approaches have been proposed to enhance the accuracy of CBFi measurement, including early time lag range (ETLR) fitting,^24^^,^^30^^,^^31^ pressure modulation,^32^ application of correction factors,^25^ depth-sensitive time-domain DCS,^33^^–^^35^ interferometric DCS,^36^^,^^37^ long wavelength DCS,^38^ and multi-channel detection.^39^^,^^40^ In parallel, analytical models have evolved from the traditional semi-infinite model to more advanced multi-layer models, including two-layer and three-layer models.^4^^,^^25^^,^^41^^–^^43^ Comparative studies have shown that multi-layer models offer advantages over the semi-infinite model in estimating relative changes in CBFi, whereas the semi-infinite model remains more sensitive to signals from superficial layers.^4^^,^^22^^,^^23^^,^^25^^,^^28^^,^^44^

However, comprehensive comparisons among different analytical models remain lacking. Although the two-layer and three-layer models have been compared with the semi-infinite model,^23^^,^^28^^,^^44^^,^^45^ direct comparisons involving all three models are limited. Zhao et al.^46^ conducted a direct comparison focusing on CBFi extraction using a clinical collected dataset; however, they did not address differences in relative CBFi (rCBFi) recovery, the influence from extracerebral layers, or the impact of errors in the assumed model parameter. Table 1 summarizes the contributions of various studies comparing analytical models and highlights the unique contributions of this work.

**Table 1:** Summary of previous studies that compared DCS analytical models. Note: a check mark (✓) indicates that the specification is included in the reference, whereas a cross mark (✗) indicates that it is not included.

Studies	Models	Data source	ρ (mm)	CBF sensitivity	Crosstalk from extracerebral BFi	Optical and physiological parameters assuming errors	Fitting methods
Gagnon et al.^4^	Semi-infinite	Two-layer slab MC simulations	10, 20, and 30	✗	✗	✓	Whole curve SD fitting
	Two-layer	MRI-scanned head model simulations					
		Two-layer liquid phantom					
Verdecchia et al.^25^	Semi-infinite	Two-layer liquid phantom	20 and 27	✗	✗	✗	Semi-infinite: ETLR fitting
						$\mu_{a}$ and $\mu_{s}^{\prime }$: TR-NIRS	
	Three-layer	Pig head experiment				Thickness: CT	Three-layer: MD fitting
Wu et al.^22^	Semi-infinite	MC simulations	5, 25, and 30	✗	✓	$\mu_{a}$ and $\mu_{s}^{\prime }$: assumed	Semi-infinite: ETLR fitting
	MC three-layer model	Human subject with hypercapnia				Thickness: MRI	
Zhao et al.^44^	Semi-infinite	Three-layer analytical model;	10 and 25	✗	✗	✓	Semi-infinite: /
	Three-layer	MC human head simulations					Three-layer: MD fitting
Forti et al.^23^	Semi-infinite	Three-layer slab MC simulations	25	✗	✗	✓	Semi-infinite: ETLR fitting
	Two-layer	Two-layer liquid phantom					
Zhao et al.^46^	Semi-infinite	Clinical dataset from patients with subarachnoid hemorrhage	10 and 25	✓	✗	✗	Semi-infinite: ETLR fitting
	Two-layer					$\mu_{a}$ and $\mu_{s}^{\prime }$: assumed	Two-layer: MD fitting
	Three-layer					Thickness: CT	Three-layer: MD fitting
Wang et al.^28^	Semi-infinite	Three-layer slab MC simulations	5, 10, 15, 20, 25, and 30	✓	✗	✓	Whole-curve SD fitting
	Three-layer						
This work	Semi-infinite	Four-layer slab MC simulations	20, 25, 30, and 35	✓	✓	✓ (short review)	Semi-infinite: ETLR fitting
	Two-layer						Two-layer: SD fitting
	Three-layer						Three-layer: MD fitting

Although previous studies have explored different models, they did not provide a quantitative comparison of crosstalk from extracerebral layers, nor did they examine crosstalk specifically originating from the skull layer BFi (BBFi). Overall, existing literature compares models in a fragmented manner, lacking comprehensiveness and offering no guidance on model selection for different experimental scenarios. Furthermore, optimal fitting strategies for these models remain unclear, and comparisons among existing fitting methods, such as single-distance (SD) and multi-distance (MD) fitting, are still needed. This article aims to address these gaps by presenting a systematic comparison of the three analytical models.

In this work, we simulate a four-layer slab human head model to generate experimental data. We investigate different fitting strategies for the three analytical models and explore their intrinsic CBFi sensitivity. We compare the accuracy of each model in recovering absolute CBFi and rCBFi across various source-detector separations (ρ=20, 25, 30, and 35 mm). We also assess how CBFi and rCBFi accuracy is influenced by assumptions regarding optical properties and the thickness of the scalp and skull, as well as by each model’s robustness to blood flow changes in extracerebral layers. In addition, we evaluate the time-to-result for each model using its respective fitting method to assess the feasibility of real-time CBFi monitoring. Finally, we discuss several key findings and current limitations of the study and provide recommendations for selecting the most appropriate model for specific applications and experimental conditions.

## 2 Methods

### 2.1 DCS Theory

The DCS theory is based on the correlation diffusion equation (CDE), derived from the correlation transfer equation (CTE) under the standard diffusion assumption.^47^^,^^48^ This derivation is analogous to the photon diffusion equation from the radiative transfer equation (RTE) using the $P_{N}$ approximation.^48^^,^^49^ The analogy between the CTE and RTE was first established by Ackerson et al.^50^ The CDE is expressed as

$$(\frac{D_{r}}{\upsilon }\nabla^{2}-\mu_{a}-\frac{1}{3}\mu_{s}^{\prime }k_{0}^{2}\alpha \langle \Delta r^{2}(\tau )\rangle )G_{1}(r,\tau )=-S(r),$$ (1)

where $G_{1}(r,\tau )=\langle E(r,t)E^{*}(r,t+\tau )\rangle$ is the electric field temporal ACF, $D_{r}=\upsilon /(3\mu_{s}^{\prime })$ is the photon diffusion coefficient, υ is the speed of light in the medium, τ is the delay time, $k_{0}=2\pi n_{0}/\lambda$ is the wavenumber of light in the scattering medium at the wavelength λ, $n_{0}$ is the tissue refractive index, $\mu_{a}$ is the absorption coefficient, $\mu_{s}^{\prime }$ is the reduced scattering coefficient, S is the source, and $\left\langle \Delta r^{2}(\tau )\right\rangle$ is the mean square displacement of scatterers. For diffusive motions, $\left\langle \Delta r^{2}(\tau )\right\rangle =6D_{B}\tau$, where $D_{B}$ is the effective Brownian diffusion coefficient. In most practical applications, the Brownian motion model is accurate to describe scatterers’ motions.^19^^,^^51^^,^^52^ The product $\alpha D_{B}$ is defined as BFi,^53^ where α is the probability of scattering from a moving scatterer, assumed to be 1 in our simulations.^54^

The normalized electric field temporal ACF, $g_{1}(\tau )$, is related to the normalized light intensity ACF, $g_{2}(\tau )$, through the Siegert equation ^55^

$$g_{2}(\tau )=1+\beta |g_{1}(\tau ){|}^{2},$$ (2)

where β is the coherence factor, which depends on the laser stability, coherence length, and the number of speckles detected.^19^ The measured light intensity ACF can be calculated as

$$g_{2}(\tau )=\frac{\left\langle I(t)I(t+\tau )\right\rangle }{\langle I(t{)\rangle }^{2}},$$ (3)

where ⟨…⟩ denotes the average over the integration time $T_{\mathrm{int}}$, and I(t) is the measured light intensity fluctuation. By fitting Eq. (3) to the analytical light intensity ACF, the BFi ($\alpha D_{B}$) can be extracted.

In this study, we consider light propagation using a commonly used continuous-wave (CW) 785-nm laser source isotropically scattered in the medium. Green’s function solution to Eq. (1), $G_{1}(r,\tau )$, has different forms under semi-infinite, two-layer, and three-layer boundary conditions. Detailed derivations of DCS analytical models can be found in the Supplementary Material.

### 2.2 Data Source

In this work, experimental data were generated using Monte Carlo (MC) simulations conducted with the voxel-based Monte Carlo eXtreme (MCX)^56^ toolkit in MATLAB (R2023b, The MathWorks). We simulated a slab human head model, with a volume of $200\times 200\times 200\;{\mathrm{mm}}^{3}$, segmented into four layers, each layer representing the scalp, skull, cerebrospinal fluid (CSF), and brain tissues; 785-nm light was used in all MC simulations in this work, and the optical parameters at 785 nm are detailed in Table 2. Tissue refraction indices were assumed as 1.37 for all layers. Each simulation was executed with $10^{9}\text{ photons}$ from a 1-mm diameter source and utilized five detectors (1 mm in diameter, positioned at ρ=15, 20, 25, 30, and 35 mm) to simultaneously record the photon transfer and photon pathlength, thereby enabling the calculation of the temporal light field ACF^53^^,^^57^

$$G_{1}(\tau )=\frac{1}{N_{p}}\overset{N_{p}}{\underset{n=1}{\sum }}\;\exp(\overset{N_{\text{tissue}}}{\underset{i=1}{\sum }}-\frac{1}{3}Y_{n,i}k_{0}^{2}{\left\langle \Delta r^{2}(\tau )\right\rangle }_{i})\exp(-\overset{N_{\text{tissue}}}{\underset{i=1}{\sum }}\mu_{a,i}L_{n,i}),$$ (4)

where $N_{p}$ is the number of detected photons at each detector; $N_{\text{tissue}}$ is the number of tissue types ($N_{\text{tissue}}=4$ in our case); $Y_{n,i}$ and $L_{n,i}$ are the total momentum transfer and total pathlength of photon n in layer i, respectively; and $\mu_{a,i}$ is the absorption coefficient in layer i. ${\left\langle \Delta r^{2}(\tau )\right\rangle }_{i}=6D_{Bi}\tau$ (see Sec. 2.1) is the mean square displacement of the scattered particles in layer i, where $D_{Bi}$ is the effective Brownian diffusion coefficient in layer i. The correlation delay time τ was adopted from our time-tagger module (SPC-QC-104, Beker & Hickl). The simulated $G_{1}(\tau )$ was normalized to $G_{1}(0)$, then $g_{2}(\tau )$ curves were obtained through Eq. (2) with β=0.5.

**Table 2:** Optical parameters at 785 nm and layer thicknesses of the four-layer human head model simulation.

	Layer	Layer thickness (mm)	$\mu_{a}$ (${\mathrm{mm}}^{-1}$)	$\mu_{s}$ (${\mathrm{mm}}^{-1}$)	g	$D_{B}$ (${\mathrm{mm}}^{2}/s$)
Four-layer slab	Scalp	5	0.019	6.600	0.89	$1\times 10^{-6}$
	Skull	7	0.014	8.600		$8\times 10^{-8}$
	CSF	2	0.001	0.002		$1\times 10^{-8}$
	Brain	∞	0.020	11.00		$6\times 10^{-6}$

We use the slab head model because it allows convenient comparison of the CBFi sensitivity by adjusting ρ, and previous research has validated that the slab model provides a reasonable approximation of the anatomically curved human head.^22^ It is known that the diffuse approximation becomes invalid in regions of low scattering, such as the CSF.^21^^,^^56^^,^^58^ Despite this, studies by Custo et al.^59^ and Zhao and Buckley^6^ indicate that the CSF has only a minor influence on brain sensitivity and rCBFi recovery. To better represent a realistic human head in our simulation, we included a 2-mm-thick CSF layer as reported by Wu et al.^30^ and Okada et al.^60^

The simulation parameters, summarized in Table 2, are based on biologically realistic tissue properties reported in previous studies at a wavelength of 785 nm.^4^^,^^21^^,^^61^^–^^64^ The reduced scattering coefficient $\mu_{s}^{\prime }$ is calculated using $\mu_{s}^{\prime }=(1-g)\mu_{s}$, where $\mu_{s}$ is the scattering coefficient and g is the tissue’s scattering anisotropy factor.^65^ To more accurately mimic the human head’s layered structure, we set the scalp and skull thicknesses to 5 and 7 mm, respectively, based on prior studies.^59^^,^^66^^,^^67^

The baseline Brownian diffusion coefficients $D_{Bi}$ (i=1, 2, 3, 4) were set as $1\times 10^{-6}$, $8\times 10^{-8}$, $1\times 10^{-8}$, $6\times 10^{-6}\;{\mathrm{mm}}^{2}/s$ for the scalp,^38^^,^^68^ skull, CSF,^6^ and brain layers,^28^^,^^30^ respectively. Notably, direct measurements of skull blood flow are limited.^32^^,^^38^ However, the skull is known to contain vascular networks, whereas the CSF layer primarily contains small perivascular capillaries.^69^ Therefore, we simulate minimal blood flow in the skull and negligible flow in the CSF layer. Specifically, $D_{B}$ values assigned to the scalp and brain layers were based on clinically reasonable relationships reported in previous studies.^30^^,^^38^^,^^68^ The simulated $g_{2}$ data from the four-layer model served as the experimental data for our analysis.

### 2.3 Noise Model

To mimic real-world DCS measurements and assess the robustness of the analytical models, we employed a Gaussian noise model with zero mean and a standard deviation based on the noise model proposed by Zhou et al.^70^ to our simulations. The standard deviation is defined as

$$\sigma (\tau )=\sqrt{\frac{T_{\text{bin}}}{T_{\mathrm{int}}}}[\beta^{2}\frac{(1+e^{-2\Gamma T_{\mathrm{bin}}})(1+e^{-2\Gamma \tau })+2m(1-e^{-2\Gamma T_{\mathrm{bin}}})e^{-2\Gamma \tau }}{1-e^{-2\Gamma T_{\text{bin}}}}+\frac{2\beta (1+e^{-2\Gamma T_{\mathrm{bin}}})}{\left\langle n\right\rangle }+\frac{1+\beta e^{-\Gamma \tau }}{{\left\langle n\right\rangle }^{2}}{]}^{1/2},$$ (5)

where Γ is the decay speed, extracted by fitting the simulated $g_{2}(\tau )$ curves with a single-exponential decay model: $g_{2}(\tau )=1+\beta \;\exp(-2\Gamma \tau )$.^70^ $T_{\text{bin}}$ is the correlator bin width, adopted from our time-tagger module. It is set to be 6.145 ns for the first 16 channels and is tripled every 16 channels thereafter. $T_{\mathrm{int}}$ is the integration time, m is the bin index, ⟨n⟩ is the average number of photons within the bin width $T_{\text{bin}}$, where $\langle n\rangle =IT_{\text{bin}}$, and I is the detected photon count rate. Gaussian noise with zero mean and a standard deviation defined by Eq. (5) was added to clean $g_{2}$ data to simulate realistic testing conditions.

The photon count rate and the integration time can influence the noise level in DCS measurements. Generally, a higher photon count rate and longer integration time yield a higher signal-to-noise ratio (SNR). However, in practice, the photon count rate is constrained by safety regulations governing maximum permissible light exposure on biological tissues, as specified in ANSI Z136.1,^71^ and it is typically not a controllable parameter for a specific ρ. In contrast, the integration time can be adjusted according to the sampling rate.^5^

In this work, we assumed a photon count rate I of 10.0 kcps at ρ=30  mm, consistent with previous reports.^38^ Photon count rates at other ρ were estimated by comparing the Green’s function solution for photon fluence at delay time zero, $G_{1}(0)$.^72^ In our simulations, the count rates were 686.1, 142.8, 35.1, 10.0, and 3.4 kcps for ρ=15, 20, 25, 30, and 35 mm, respectively. The integration time $T_{\mathrm{int}}$ was set to 60 s. The SNR of the synthesized $g_{2}$ was calculated using^73^^,^^74^

$$\mathrm{SNR}=(g_{2}(\tau )-1)/\sigma (\tau ).$$ (6)

We have validated the noise model using measurements on a milk phantom; the validation results are provided in Fig. S2 in the Supplementary Material. For our simulations, 20 noise realizations were added to each CBFi condition. The resulting synthetic noisy dataset was used to evaluate the performance of the analytical models under realistic experimental conditions.

### 2.4 Fitting Methods

For the semi-infinite analytical model, previous studies have shown that ETLR fitting can enhance CBF sensitivity, although this improvement comes at the cost of reduced SNR.^24^^,^^30^^,^^31^ For the three-layer model, Zhao et al.^44^ and Verdecchia et al.^25^ employed MD fitting to obtain optimized solutions. In this study, we apply ETLR fitting to the semi-infinite model and evaluate both MD and SD fitting approaches for the multi-layer models.

#### 2.4.1 Penalty function for the semi-infinite fitting

**Fig. 1:**
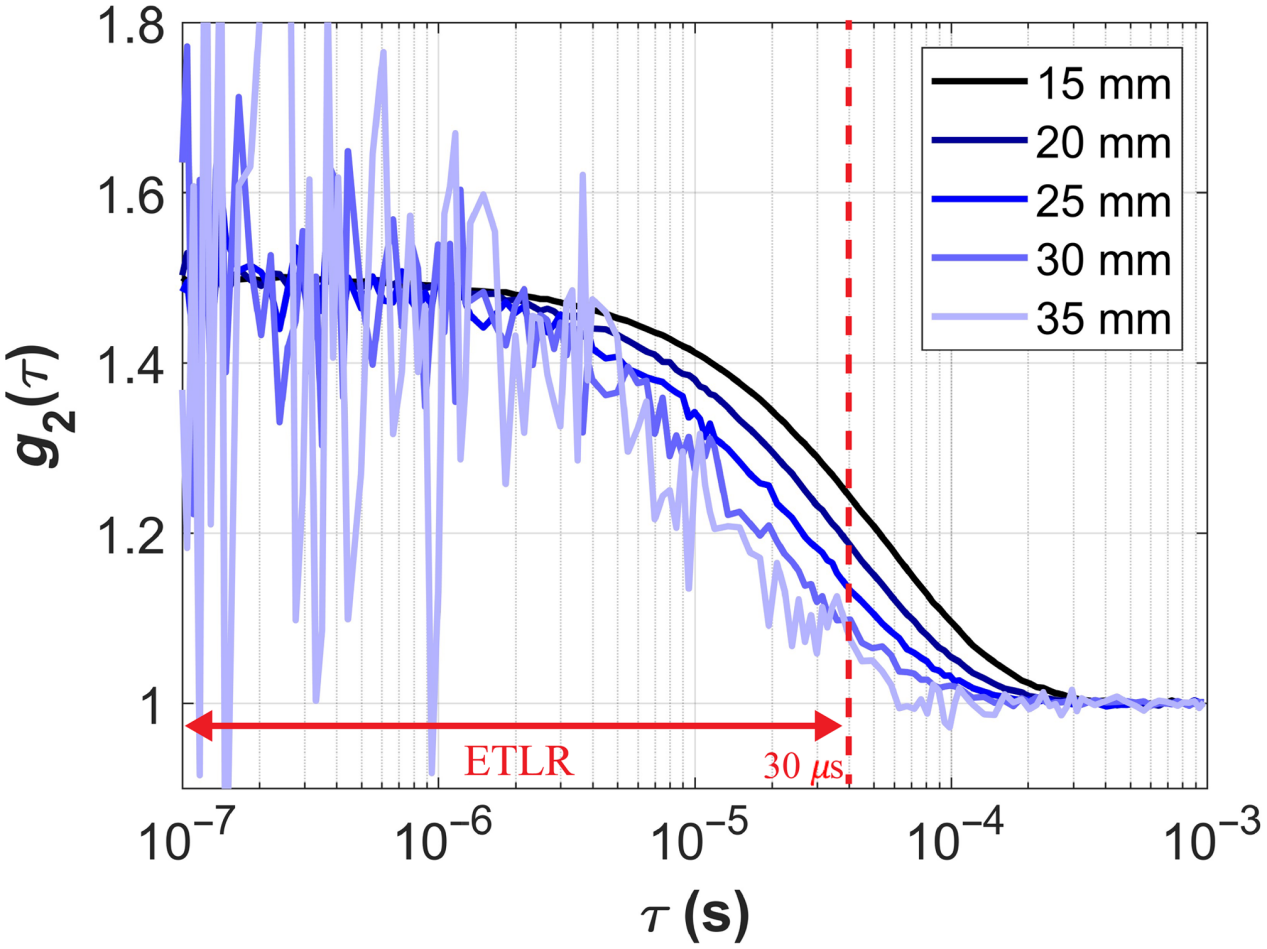
$g_{2}$ curves at different ρ; the red line with arrows shows the ETLR window.

For ETLR fitting, previous studies have constrained $g_{2}\geq 1.25$.^22^^,^^75^ In this work, to facilitate our analysis, we simply restrict the ETLR to τ≤30  μs across different ρ^76^ as shown in Fig. 1. We use the *fminsearchbnd* function in MATLAB to minimize the single penalty function defined as

$$\chi^{2}=\overset{N_{\tau }}{\underset{i=1}{\sum }}[g_{2}^{\text{theory}}(\rho ,\tau_{i},{\mathrm{CBFi}}_{\text{estimated}})-g_{2}^{\text{simulated}}(\rho ,\tau_{i},{\mathrm{CBFi}}_{\text{true}}){]}^{2},$$ (7)

where $N_{\tau }$ is the number of τ, and $\tau_{i}$ is the i’th delay time. CBFi is the fitting parameter, constrained within the bounds $\in [10^{-9},10^{-4}]\;{\mathrm{mm}}^{2}/s$. We extracted CBFi at ρ=15, 20, 25, 30, and 35 mm. The optical parameters for the semi-infinite fitting were assumed to be known and taken from the brain layer, as listed in Table 2. In previous studies, CBFi and β were simultaneously estimated through fitting.^28^^,^^77^ However, treating β as a fitting parameter tends to reduce the convergence and stability of the estimated CBFi. In this work, we compare semi-infinite ETLR fitting with and without setting β as a fitting parameter.

As the use of single-exponential fitting for processing DCS data to estimate rCBFi has become increasingly common, it is important to note that the reliability of this method has not yet been validated against the semi-infinite analytical fitting. It is known that the Green’s function solution to the CDE under a semi-infinite geometry can be simplified to a single-exponential decay function at short correlation times,^70^^,^^78^^,^^79^ as is introduced in Sec. 2.3. Under this approximation, the normalized electric field ACF is expressed as $g_{1}(\tau )=e^{-\tau /\tau_{c}}$. According to Siegert’s equation,^55^ the normalized light intensity ACF is then given by $g_{2}(\tau )=1+\beta |g_{1}(\tau ){|}^{2}$, i.e.

$$g_{2}(\tau )=1+\beta e^{-\frac{2\tau }{\tau_{c}}},$$ (8)

where $\tau_{c}$ is the decorrelation time. In this work, we also compare the single-exponential fitting using Eq. (8) with the semi-infinite ETLR analytical fitting. The penalty function for single-exponential fitting is defined as

$$\chi^{2}=\overset{N_{\tau }}{\underset{i=1}{\sum }}[g_{2}^{\text{exponential}}(\rho ,\tau_{i},\tau_{c})-g_{2}^{\text{simulated}}(\rho ,\tau_{i},{\mathrm{CBFi}}_{\text{true}}){]}^{2}.$$ (9)

The reciprocal of $\tau_{c}$, referred to as the decorrelation speed, is used to calculate rCBFi (rCBFi = $\tau_{c\_\text{baseline}}/\tau_{c}$). The *fminsearchbnd* function was used to minimize the penalty function in MATLAB. This result was then compared with rCBFi recovered from the semi-infinite ETLR analytical fitting.

#### 2.4.2 Penalty function for multi-layer models fitting

In addition to fitting a single $g_{2}$ curve, we also implemented MD fitting for the two- and three-layer models. Namely, we simultaneously fit two $g_{2}$ curves, one is at a smaller ρ (ρ=15  mm), and another is larger ρ (ρ=20, 25, 30, and 35 mm). MD fitting has been demonstrated to improve CBFi recovery accuracy when using multi-layer DCS models.^57^ The penalty function for multi-layer models fitting is defined as

$$\chi^{2}=\overset{N_{\rho }}{\underset{j=1}{\sum }}\overset{N_{\tau }}{\underset{i=1}{\sum }}[g_{2}^{\text{theory}}(\rho_{j},\tau_{i},{\mathrm{SBFi}}_{\text{recovered}},{\mathrm{CBFi}}_{\text{recovered}})-g_{2}^{\text{simulated}}(\rho_{j},\tau_{i},{\mathrm{SBFi}}_{\text{true}},{\mathrm{CBFi}}_{\text{true}}){]}^{2},$$ (10)

where $\rho_{j}$ is the j’th ρ, and $N_{\rho }=1$ for SD multi-layer fitting (= 2 for MD multi-layer fitting). SBFi and CBFi are the fitting parameters; we use the *fminsearchbnd* function to minimize $\chi^{2}$ and set the bounds for SBFi and CBFi both $\in [10^{-9},10^{-4}]{\mathrm{mm}}^{2}/s$. As in previous cases, the optical and physiological parameters (e.g., layer thicknesses) were assumed to be known prior to fitting, as detailed in Table 2. For the three-layer fitting, the first and second layers represent the scalp and skull layers of the four-layer slab head model. The CSF layer is neglected and grouped with the cerebral layer, following the approach reported in a previous study.^6^ For the two-layer fitting, the extracerebral optical parameters are taken from the scalp layer of the four-layer model, and the extracerebral layer thickness is defined as the combined thickness of the scalp and skull layers, again, with the CSF included in the cerebral layer.

For MD fitting, β was treated as a fitting parameter with bounds ∈ [0, 1]; for SD fitting, β was either fixed at 0.5 or treated as a fitting parameter. We compared MD and SD fitting strategies for the multi-layer analytical models and, based on this comparison, selected the optimal fitting approach for CBFi recovery in subsequent assessments using the multi-layer models.

### 2.5 CBFi Sensitivity, Absolute CBFi, and rCBFi Recovery

To evaluate the sensitivity of different analytical models to changes in CBFi, we simulated CBF perturbations in layer 4 of the four-layer model. Specifically, $D_{B}$ in layer 4 (brain layer) was varied by ±25% and ±50% relative to the baseline ($D_{B0}=6\times 10^{-6}\;{\mathrm{mm}}^{2}/s$) while keeping $D_{B}$ in Layer 1, 2, and 3 constants (i.e., $D_{B1,2,3}=1\times 10^{-6}$, $8\times 10^{-8}$, $1\times 10^{-8}\;{\mathrm{mm}}^{2}/s$). As described in Sec. 2.3, 20 noise realizations were added to each CBFi condition, resulting in 100 samples for each ρ value (20 realizations × 5 conditions, including one baseline and four perturbation levels). The mean CBFi value recovered under the baseline condition is used as the global baseline for calculating rCBFi, defined as^80^

$$\mathrm{rCBFi}=\frac{{\mathrm{CBFi}}_{\text{recovered}}}{{\mathrm{CBFi}}_{0\_\text{recovered}}},$$ (11)

where ${\mathrm{CBFi}}_{\text{recovered}}$ and ${\mathrm{CBFi}}_{0\_\text{recovered}}$ are the recovered CBFi at perturbed and baseline conditions, respectively. The generated perturbed $g_{2}$ data were then used to extract absolute SBFi, CBFi, and rCBFi and calculate the CBFi sensitivity defined as^24^^,^^63^

$$S_{\mathrm{CBFi}}=\frac{({\mathrm{CBFi}}_{\text{recovered}}/{\mathrm{CBFi}}_{0\_\text{recovered}})-1}{(D_{B4}/D_{B0,4})-1}\times 100\%,$$ (12)

where $D_{B4}$ and $D_{B0,4}$ are the simulated perturbed and baseline CBFi. A negative sensitivity indicates that the model responds inversely to changes in CBFi, whereas a sensitivity greater than 100% suggests that the model overreacts, amplifying the effect of CBFi changes.

### 2.6 Optical and Physiological Parameters Assumption Errors

Typically, when using DCS to extract CBFi, optical parameters ($\mu_{a}$ and $\mu_{s}^{\prime }$) and layer thicknesses are adopted from values reported in previous studies.^4^^,^^44^ However, incorrect assumptions about these parameters can introduce significant inaccuracies in CBFi estimation. As the impact of parameter assumption errors on all three analytical models has already been examined in prior publications, we provide only a brief review of this topic in the present work and do not perform a quantitative analysis.

### 2.7 Crosstalk from Extracerebral BF

The primary confounder in CBFi measurements using DCS is the blood flow in the extracerebral layer.^81^ SBFi constitutes a significant portion of cerebral perfusion, whereas skull blood flow, though often overlooked, is also substantial due to its vascular networks and considerable thickness. We argue that assuming zero blood flow in the skull layer when using multi-layer DCS models to recover CBFi is not a good approximation. To investigate this, we varied the skull BFi (BBFi) while keeping blood flow in other layers constant, to study the correlation between recovered CBFi and BBFi variations across different analytical models.

In this section, we characterized the crosstalk raised by the variations in SBFi and BBFi separately. Specifically, we use the four-layer model to simulate $g_{2}$ data by varying the Brownian diffusion coefficients in the scalp layer varied across five levels: $2\times 10^{-7}$, $6\times 10^{-7}$, $1\times 10^{-6}$, $1.4\times 10^{-6}$, and $1.8\times 10^{-6}\;{\mathrm{mm}}^{2}/s$ while keeping the Brownian diffusion coefficients in other layers fixed at baseline values (Table 2). Similarly, to assess the effect of BBFi variations, the Brownian diffusion coefficient in the skull layer was set to five levels: 0, $4\times 10^{-8}$, $8\times 10^{-8}$, $1.2\times 10^{-7}$, and $1.6\times 10^{-7}\;{\mathrm{mm}}^{2}/s$ while keeping baseline Brownian diffusion coefficients in the other layers. We use the fitting methods described in Sec. 2.4 to recover CBFi. To evaluate the sensitivity of the recovered CBFi to variations in extracerebral blood flow, we calculated sensitivities using Eq. (12), where $D_{B4}$ and $D_{B0,4}$ were replaced with the simulated SBFi and BBFi values under perturbed and baseline conditions, respectively.

### 2.8 Time to Results

To evaluate the capability of different models for real-time CBF monitoring, we compared the computational time required by the semi-infinite, two-layer, and three-layer models using their respective fitting strategies. First, we compared ETLR fitting with single-exponential fitting for the semi-infinite model. Second, we assessed MD fitting versus SD fitting for the multi-layer models. Finally, we compared all fitting strategies across the three models and discussed their feasibility for real-time CBF monitoring.

## 3 Results

### 3.1 Fitting Methods

**Fig. 2:**
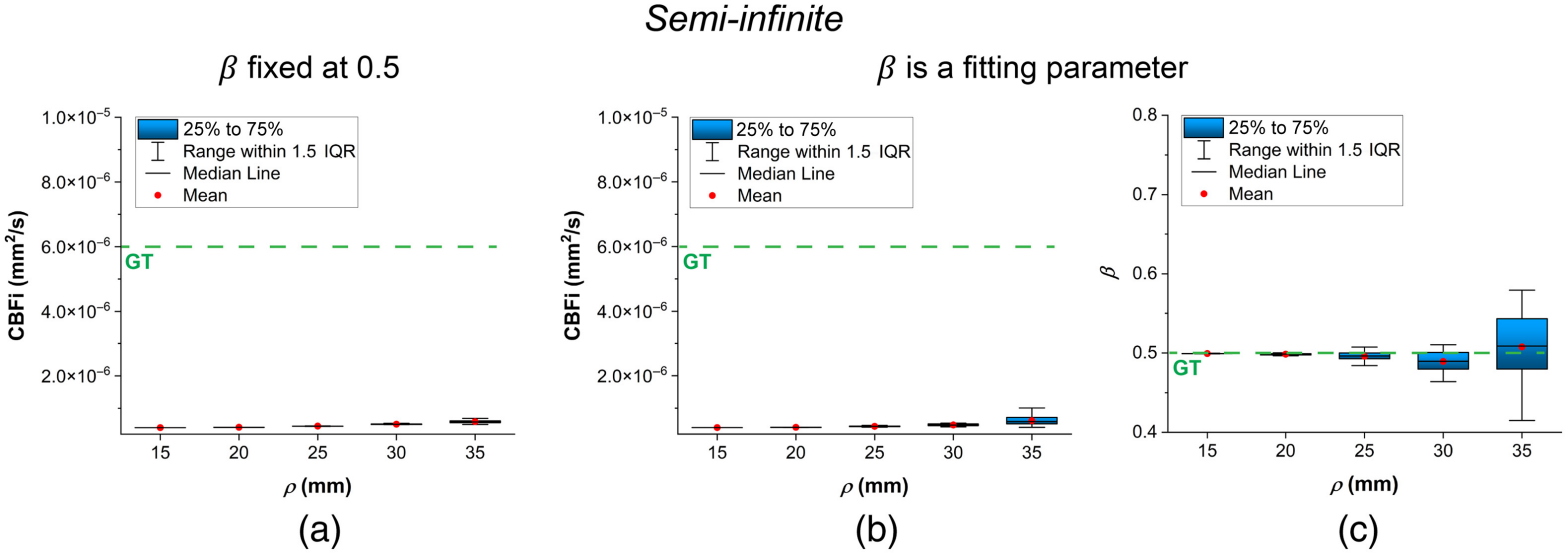
Semi-infinite model recovered CBFi at different ρ. (a) CBFi recovered using ETLR fitting, β was fixed at 0.5. (c) CBFi recovered using ETLR fitting, whereas β was treated as a fitting parameter. (d) Recovered β at different ρ. The green dashed lines with “GT” represent parameter ground truths.

**Fig. 3:**
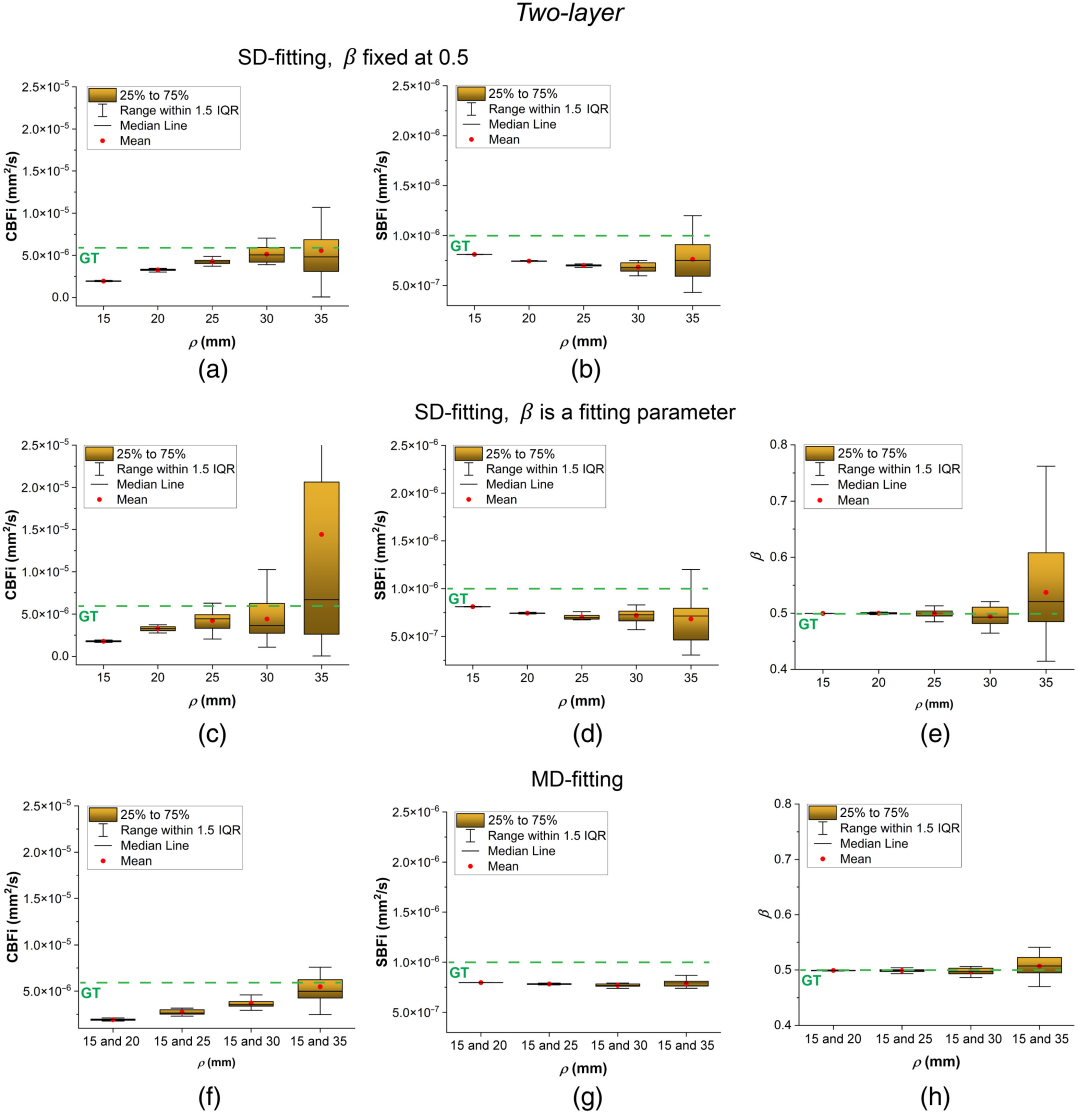
Two-layer model fitting results at different ρ. (a) and (b) Recovered CBFi and SBFi using SD fitting as β=0.5. (c)–(e) Recovered CBFi, SBFi, and β using SD fitting as β was treated as a fitting parameter. (f)–(h) Recovered CBFi, SBFi, and β using MD fitting. The green dashed lines with “GT” represent parameter ground truths.

**Fig. 4:**
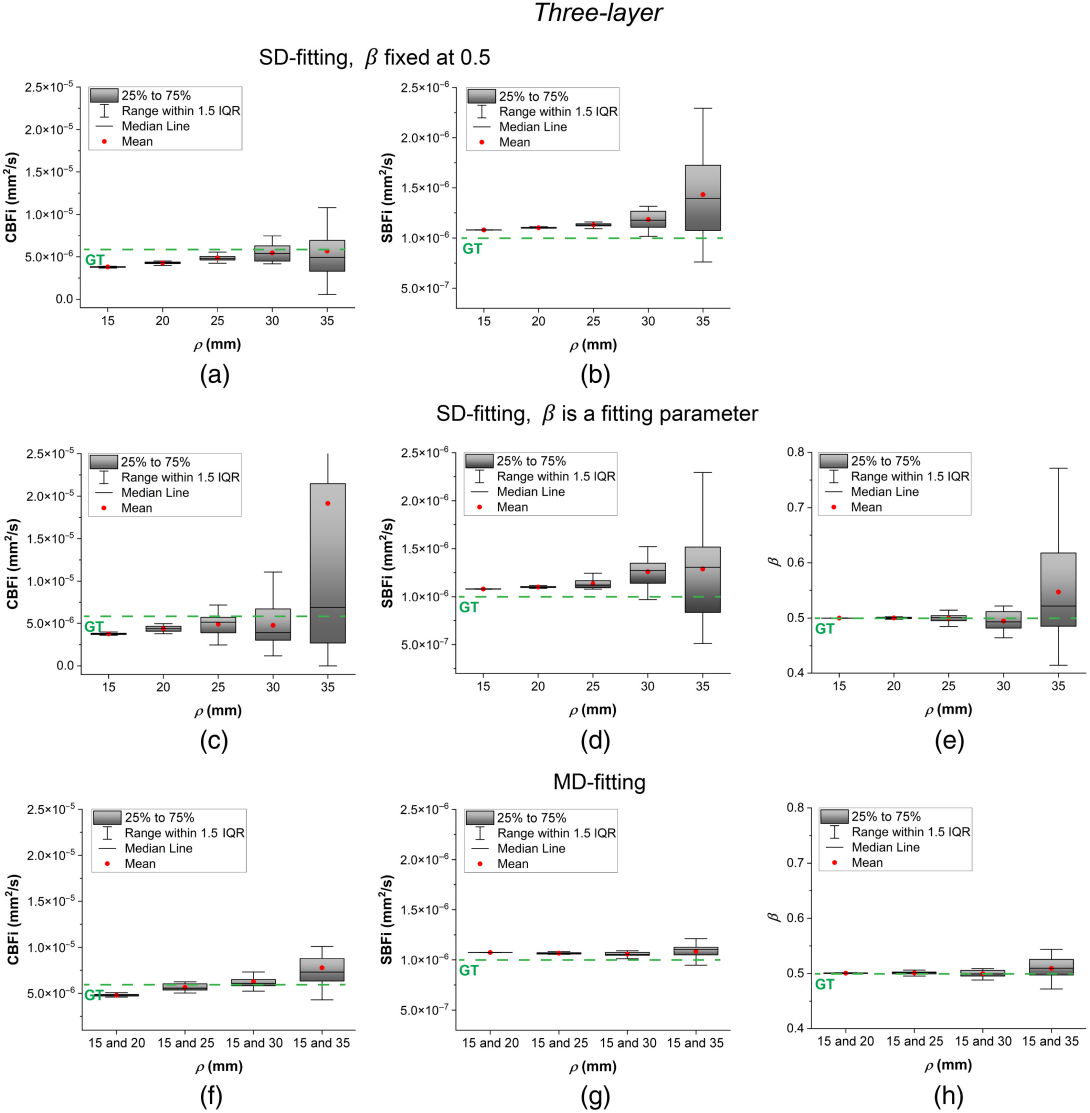
Three-layer model fitting results at different ρ. (a) and (b) Recovered CBFi and SBFi using SD fitting while β=0.5. (c)–(e) Recovered CBFi, SBFi, and β using SD fitting while β was treated as a fitting parameter. (f)–(h) Recovered CBFi, SBFi, and β using MD fitting. The green dashed lines with “GT” represent parameter ground truths.

In this section, we present the results of our comparison of different fitting methods for the three analytical models. The results are illustrated using box plots with error bars in Figs. 2–4. In these plots, each box represents the interquartile range (25% to 75%) of the recovered values (with a total of 20 samples for each parameter). The median is shown as a horizontal line inside the box, and the mean is indicated by a red point. The whiskers extend to 1.5 times the interquartile range, allowing identification of potential outliers.

Figure 2 shows the results of semi-infinite ETLR fitting, comparing cases where β is fixed versus β treated as a fitting parameter. Both approaches significantly underestimate CBFi; however, fixing β as a constant yields more stable estimates than allowing it to vary during fitting. The variability, indicated by larger error bars, increases with larger ρ values. In addition, β can be accurately recovered using semi-infinite ETLR fitting.

Figure 3 illustrates the performance of three fitting strategies for the two-layer analytical model: SD fitting with β fixed, SD fitting with β as a fitting parameter, and MD fitting. For SD fitting, treating β as a fitting parameter results in large fluctuations particularly at larger ρ values (≥30  mm), leading to instability in the estimated BFi. In contrast, MD fitting yields relatively stable estimates of CBFi, SBFi, and β, although both CBFi and SBFi are slightly underestimated. Notably, SD fitting with β fixed produces CBFi estimates that are closer to the ground truth than those obtained with MD fitting.

Figure 4 presents the results for the three-layer analytical model using both SD and MD fitting. The recovered CBFi and SBFi values are closer to the ground truth than those obtained from the semi-infinite or two-layer models, although there is a slight tendency to underestimate CBFi and overestimate SBFi. Among all methods, three-layer MD fitting provides the most accurate and stable estimates of CBFi and SBFi. In contrast, three-layer SD fitting with β as a fitting parameter exhibits significant divergence and should be approached with caution due to concerns about the reliability of the recovered results.

Based on these results, we conclude that ETLR fitting with β fixed is optimal for the semi-infinite model, SD fitting with β fixed is preferable for the two-layer model, and MD fitting is best suited for the three-layer model. Therefore, we will adopt these respective fitting methods for further analyses in the following sections.

### 3.2 rCBFi Recovery

**Fig. 5:**
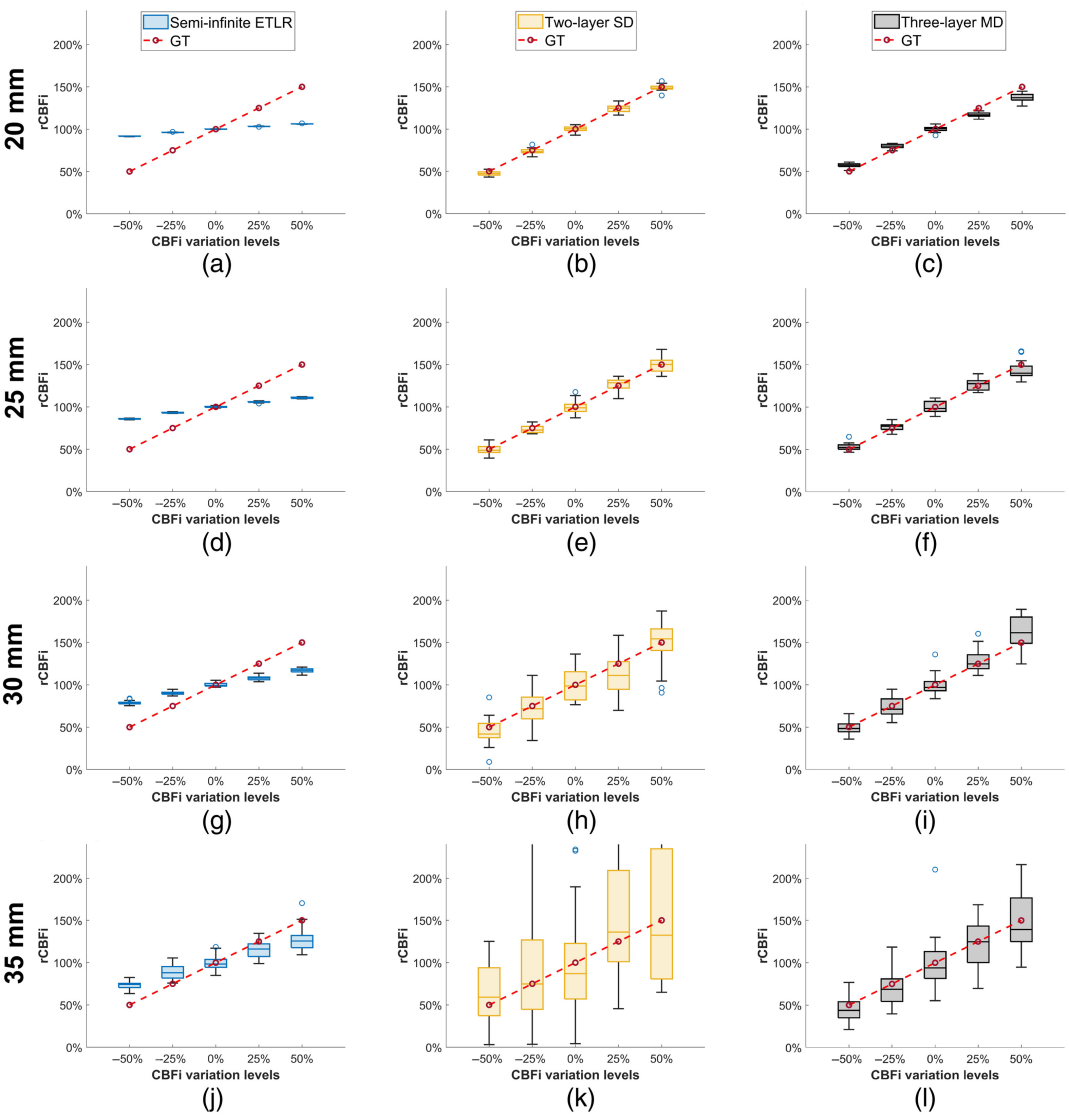
rCBFi recovered using semi-infinite ETLR fitting, two-layer SD fitting, and three-layer MD fitting at different ρ. The simulated CBFi perturbation has four levels, ±50% and ±25% compared with the baseline ($6\times 10^{-6}\;{\mathrm{mm}}^{2}/s$). We simulated 20 samples for each level at ρ=20, 25, 30, and 35 mm.

In this section, we compare the recovered rCBFi (ρ=20, 25, 30, and 35 mm) for the three analytical models using their corresponding fitting methods. Figure 5(a) shows that rCBFi values recovered using a semi-infinite ETLR fitting approach the ground truth as ρ increases, although all estimates remain underestimated. In contrast, both two-layer SD fitting and three-layer MD fitting accurately recover rCBFi across all ρ values, as shown in Figs. 5(b) and 5(c). However, at ρ=35  mm, the rCBFi estimates from two-layer SD fitting exhibit substantial fluctuations, making those results less reliable. Meanwhile, three-layer MD fitting demonstrates more consistent convergence at a larger ρ.

We also analyzed the performance of two-layer MD fitting for rCBFi recovery. Two-layer MD fitting tends to overestimate rCBFi across all ρ values, with results presented in Fig. S3 in the Supplementary Material.

### 3.3 CBFi Sensitivity

**Fig. 6:**
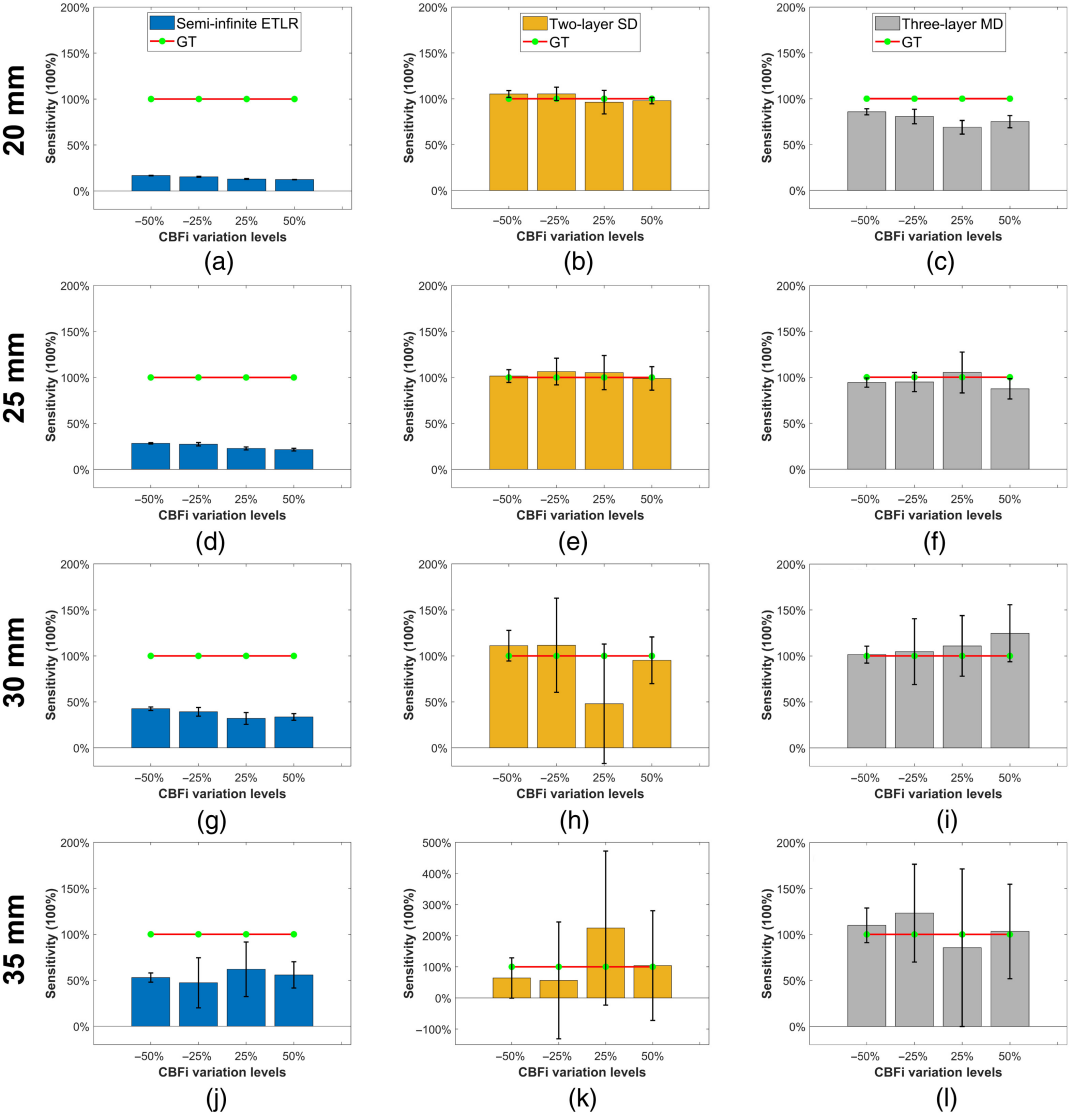
CBFi sensitivity using semi-infinite ETLR fitting, two-layer SD fitting, and three-layer MD fitting at different ρ (20, 25, 30, and 35 mm). The simulated CBFi perturbation has four levels, ±50% and ±25% compared with the baseline ($6\times 10^{-6}\;{\mathrm{mm}}^{2}/s$). CBFi sensitivity was calculated using Eq. (12).

To evaluate the sensitivity of different models to the changes in brain blood flow, we compared CBFi sensitivity using Eq. (12) on the same dataset used for rCBFi estimation. Figure 6(a) shows that the CBFi sensitivity of semi-infinite ETLR fitting increases with larger ρ values, reaching ∼50% at 35 mm. Figures 6(b) and 6(c) present the CBFi sensitivities for two-layer SD fitting and three-layer MD fitting, respectively. Both models exhibit comparable overall CBFi sensitivities, but differ in stability: at ρ=30 and 35 mm, the sensitivities show substantial fluctuations, indicating that variations in response magnitude could introduce errors in CBFi estimation. Notably, two-layer SD fitting occasionally exhibits negative sensitivity values, which, according to Eq. (12), imply a negative response to changes in CBFi.

### 3.4 CBFi Sensitivity to Extracerebral BFi Changes

**Fig. 7:**
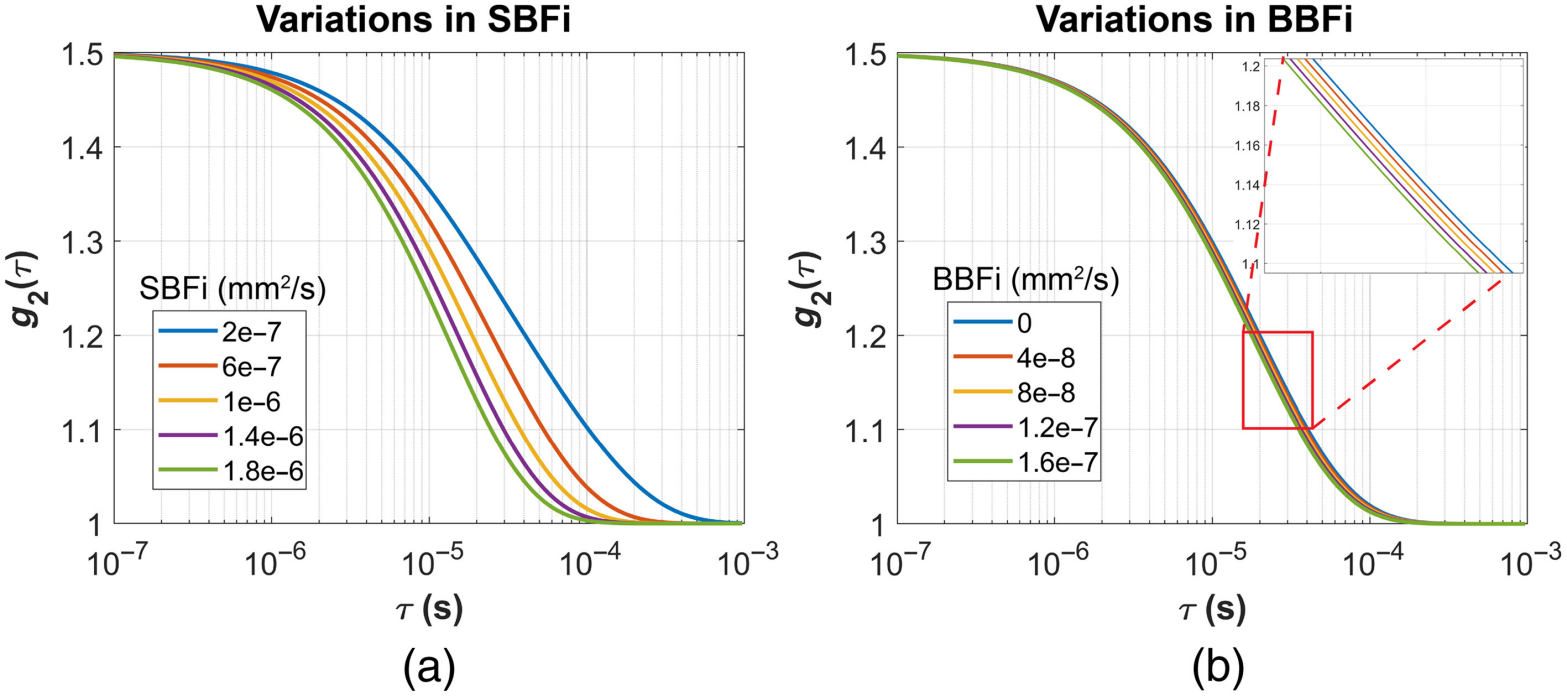
(a) and (b) Simulated $g_{2}$ curves with variations in SBFi and BBFi at ρ=30  mm.

In this section, we compare the CBFi sensitivity of the three analytical models to variations in extracerebral BFi. Five different levels of SBFi and BBFi were simulated using the four-layer head model, as introduced in Sec. 2.7. Figures 7(a) and 7(b) show the simulated noise-free $g_{2}$ curves corresponding to variations in SBFi and BBFi, respectively. Changes in both SBFi and BBFi primarily affect the latter portion of the $g_{2}$ curves. However, variations in SBFi result in more pronounced alterations in curve shape, as SBFi is substantially larger in magnitude than BBFi.

#### 3.4.1 Variations in SBFi

**Fig. 8:**
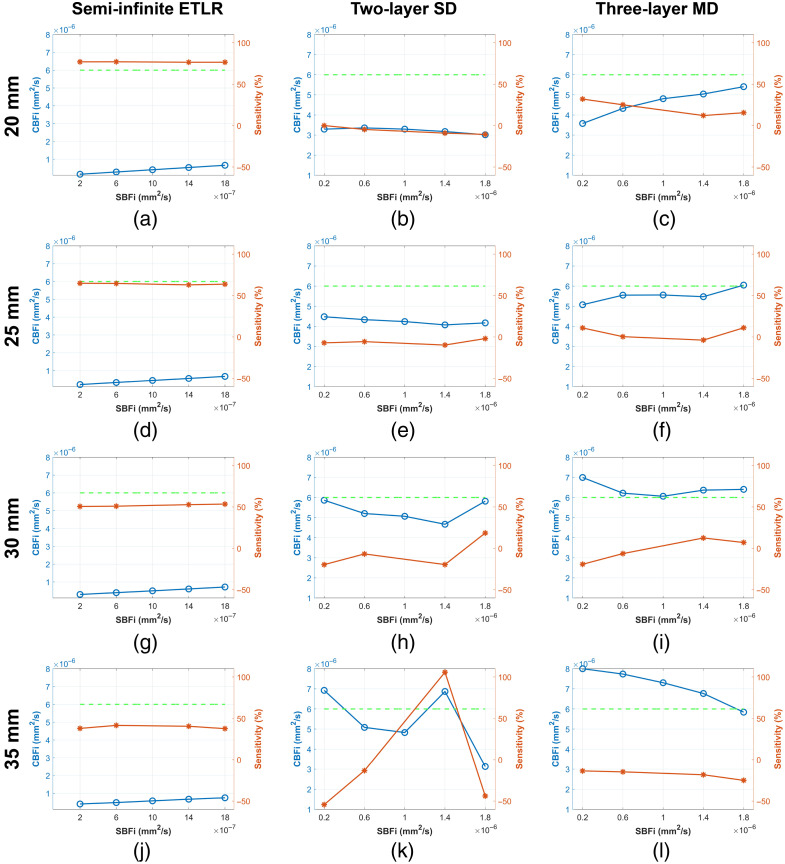
(a)–(d) Semi-infinite ETLR fitting. (e)–(h) Two-layer SD fitting. (i)–(l) Three-layer MD fitting recovered CBFi and CBFi sensitivity to SBFi variations across four different ρ (20, 25, 30, and 35 mm). The blue lines are the recovered CBFi at five different SBFi; the orange lines are the sensitivities calculated using Eq. (12); the dashed green line denotes the simulated CBFi ground truth. Note that for each variation, we simulated 20 samples; the plotted results are the median for the 20 samples to illustrate the tendency.

Figure 8 illustrates the CBFi sensitivity of different models to variations in SBFi across various ρ (20, 25, 30, and 35 mm). As shown in Fig. 8(a), the semi-infinite model exhibits positive CBFi sensitivity to SBFi variations at all ρ values, with sensitivity decreasing as ρ decreases. Consequently, the recovered CBFi increases with increasing ρ. For the two-layer model, sensitivities remain close to zero across all ρ values, resulting in CBFi estimates that are relatively stable with respect to SBFi variations. However, the sensitivity fluctuates considerably at larger ρ, leading to instability in the estimated CBFi under SBFi variations. In contrast, the three-layer model shows predominantly positive sensitivities at ρ=20 and 25 mm, resulting in increased recovered CBFi values under SBFi variations. At ρ=35  mm, however, the sensitivity becomes negative, leading to decreased recovered CBFi. Among all tested distances, the three-layer model demonstrates relatively more stable CBFi recovery at ρ=30  mm.

#### 3.4.2 Variations in BBFi

**Fig. 9:**
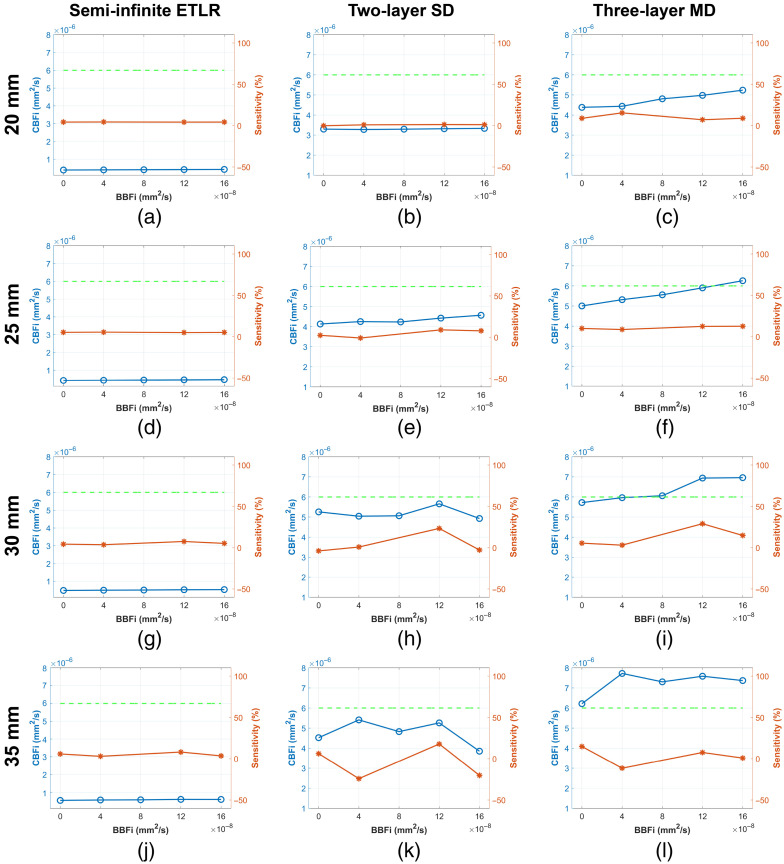
(a)–(d) Semi-infinite ETLR fitting. (e)–(h) Two-layer SD fitting. (i)–(l) Three-layer MD fitting recovered CBFi and CBFi sensitivity to BBFi variations across four different ρ (20, 25, 30, and 35 mm). The blue lines are the recovered CBFi at five different BBFi; the orange lines are the sensitivities calculated using Eq. (12); the dashed green line denotes the simulated CBFi ground truth. Note that for each variation, we simulated 20 samples; the plotted results are the median for the 20 samples to illustrate the tendency.

Figure 9 illustrates the CBFi sensitivity of different models to variations in BBFi across various ρ (20, 25, 30, and 35 mm). As shown in Figs. 9(a) and 9(b), both the semi-infinite and two-layer models exhibit sensitivities close to zero, resulting in stable recovered CBFi across all ρ values. In contrast, the three-layer model tends to display positive sensitivity at ρ=20, 25, and 30 mm, leading to an observed increase in recovered CBFi under BBFi variations.

### 3.5 Model Parameters Assumption Error

It is clear that errors in assumed model parameters can lead to inaccuracies in the estimated absolute CBFi. Several studies have quantitatively addressed this issue, listed in Table 1. Table 3 summarizes the parameters typically assumed for the three analytical models. For the semi-infinite model, the commonly assumed parameters are $\mu_{a}$ and $\mu_{s}^{\prime }$. Previous reports^28^^,^^51^ have quantitatively examined their influence, showing that $\mu_{a}$ has a positive effect on CBFi estimation, whereas $\mu_{s}^{\prime }$ has a negative effect on CBFi estimation.

**Table 3:** Pre-known and fitting parameters for each model using their respective fitting methods.

Methods	Preknown parameters	Fitting parameters
Semi-infinite ETLR	β, $\mu_{a}$, and $\mu_{s}^{\prime }$	$D_{b}$
Single-exponential	β	$\tau_{c}$
Two-layer SD	β, $\mu_{a1}$, $\mu_{s1}^{\prime }$, $\mu_{a2}$, $\mu_{s2}^{\prime }$, and $L_{ex\mathrm{tra}}$	$D_{b\_\text{extra}}$ and $D_{b\_\text{brain}}$
Three-layer MD	$\mu_{a1}$, $\mu_{s1}^{\prime }$, $\mu_{a2}$, $\mu_{s2}^{\prime }$, $\mu_{a3}$, $\mu_{s3}^{\prime }$, $L_{\text{scalp}}$, and $L_{\text{skull}}$	β, $D_{b\_\text{scalp}}$, and $D_{b\_\text{brain}}$

For the two-layer model, our previous work^82^ demonstrated that brain $\mu_{a}$ has a positive relationship with CBFi estimation error, whereas $\mu_{s}^{\prime }$ has a negative relationship. Moreover, the thickness of the extracerebral layer is positively associated with CBFi estimation error. Similarly, for the three-layer model, Zhao et al.^44^ reported relationships consistent with those found for the two-layer model. It is worth noting that for both the two-layer and three-layer models, errors in assumed model parameters do not affect the recovered rCBFi, as reported by Pan et al.^82^ and Zhao et al.,^44^ respectively.

### 3.6 Comparison Between Semi-Infinite ETLR and Single-Exponential Fitting

**Fig. 10:**
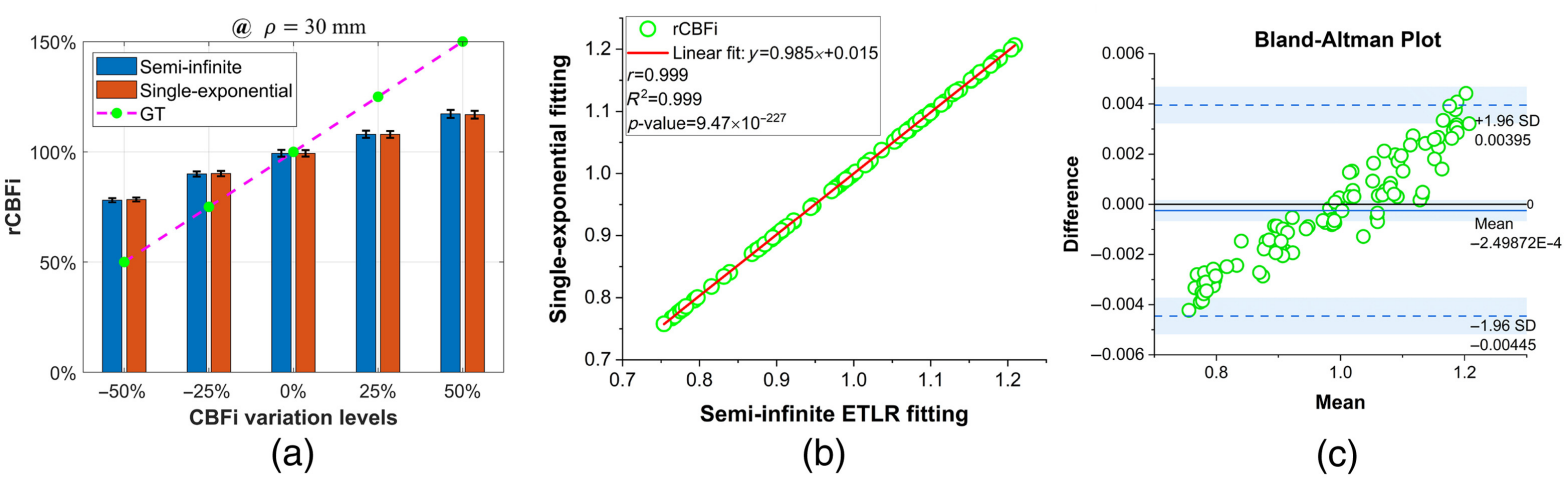
Comparison of rCBFi estimated by the semi-infinite analytical fitting and single-exponential fitting at ρ=30  mm. The *fminsearchbnd* function was used in MATLAB to minimize the penalty functions. (a) Bars represent the median rCBFi values, and the error bars indicate the interquartile range (25th to 75th percentiles). (b) Scatter plot of rCBFi values obtained with the two methods, along with the linear regression line. (c) Bland–Altman plot showing the agreement between the two methods across five different CBFi levels (total of 500 samples).

We compared and validated the effectiveness of using single-exponential fitting as a replacement for semi-infinite analytical fitting in rCBFi estimation. As shown in Fig. 10(a), single-exponential fitting demonstrates comparable accuracy and robustness in recovering rCBFi. The Pearson correlation analysis in Fig. 10(b) shows that the two methods are strongly correlated (r=0.999, $R^{2}=0.999$, p<0.0001). Besides, Bland–Altman analysis reveals strong agreement between the two methods [Fig. 10(c)], suggesting that single-exponential fitting can serve as a simple and effective alternative for rCBFi estimation.

It is worth noting that we also applied the *lsqcurvefit* function, which is based on the Levenberg–Marquardt algorithm, to minimize the penalty function defined in Eq. (9) for single-exponential fitting. However, the rCBFi recovered using *lsqcurvefit* showed greater fluctuations and more underestimation compared with results obtained using the *fminsearchbnd* function (see Fig. S4 in the Supplementary Material). This finding suggests that *lsqcurvefit* may not be suitable for this task, and researchers should carefully consider the choice of optimization method when applying single-exponential fitting.

### 3.7 Time-to-Result Comparison

As defined in the penalty functions for the chosen fitting strategies for the semi-infinite [Eq. (7)], two-layer, and three-layer [Eq. (10)] models, the required predefined and fitted parameters are listed in Table 3. Table 4 summarizes the number of model parameters and the associated computational time required for fitting, based on the dataset used for rCBFi recovery, which includes a total of 500 data samples.

**Table 4:** Time cost for different models.

Models	Number of Preknown parameters	Number of fitting parameters	Number of $g_{2}$ samples	Fitting methods	Optimization method	Time (s)
Semi-infinite	3	1	500	ETLR	*fminsearchbnd*	0.38
Single-exponential	1	1		ETLR		0.28
Two-layer	6	2		SD		9502.18
Three-layer	8	3		MD		35,099.34

The semi-infinite and single-exponential models are clearly the fastest, processing 500 data samples in just 0.38 and 0.28 s, respectively. In contrast, the two-layer model requires 9502.18 s, whereas the three-layer model takes 35,099.34 s using their respective fitting methods.

## 4 Discussion

We systematically compared three DCS analytical models: the semi-infinite, two-layer, and three-layer models, in terms of CBFi sensitivity, accuracy of absolute CBFi and rCBFi recovery, ability to separate confounding signals from extracerebral layers, sensitivity to errors in assumed model parameters, and time-to-result. Through these comparisons, we identified several findings that, to our knowledge, have not been previously reported and are important considerations for researchers selecting or applying these models in different contexts.

First, we examined the different fitting strategies for these models. For the semi-infinite model, we used ETLR fitting, as our primary goal was to assess performance in CBFi recovery. ETLR fitting can partially suppress contributions from superficial blood flow, thereby enhancing CBFi sensitivity. As shown in Fig. 2, the semi-infinite model consistently underestimates true CBFi, although the recovered CBFi increases with larger ρ values. We also compared semi-infinite ETLR fitting with and without treating β as a fitting parameter. Allowing β to vary during fitting led to greater fluctuations in the results compared with fixing it as a constant. β can be estimated by averaging the initial values of the curve at short τ and with long integration times. We recommend that researchers fix β as a constant during semi-infinite ETLR fitting, as this approach yields more stable CBFi estimates, particularly because ETLR can reduce the SNR.^30^

We also investigated different fitting methods for the two-layer and three-layer models. As shown in Fig. 3, two-layer SD fitting with β fixed provides more robust CBFi estimates across different ρ values than fitting with β as a free parameter. Although two-layer MD fitting offers the most robust estimation of all fitting parameters across the full range of ρ, it tends to deviate further from the ground truth due to model bias, particularly at smaller ρ. In contrast, the three-layer MD fitting delivered the most robust and accurate estimates of all fitting parameters, with values closely matching the ground truth, as illustrated in Fig. 4. This demonstrates that the three-layer model can better capture the layered structure of the human head and is suitable for recovering both CBFi and SBFi simultaneously using MD fitting.

Despite the promising performance of multi-layer models in CBFi recovery, it is important to recognize that they require more predefined model parameters for fitting, as summarized in Tables 3 and 4. When these parameters are inaccurately assumed, large errors in CBFi estimation can occur, particularly for the brain layer.^28^^,^^44^ Therefore, researchers should carefully select the most suitable model based on their ability to reliably obtain the necessary parameters, as well as the fitting strategies discussed above.

However, in many DCS applications, rCBFi estimation is preferred,^7^^,^^83^ and the errors due to assumed model parameters are minimized, as reviewed in Sec. 3.5. Currently, the semi-infinite model is the most widely used approach for rCBFi estimation.^9^^,^^10^^,^^12^^,^^15^ As shown in Fig. 5, the semi-infinite model’s recovered rCBFi approaches the ground truth as ρ increases and provides the most robust rCBFi estimates. These results indicate that the semi-infinite model can effectively recover rCBFi at larger ρ values, whereas both the two-layer and three-layer models can effectively recover rCBFi across all ρ. In addition, we validated single-exponential ETLR fitting and compared it to semi-infinite ETLR fitting at ρ=30  mm, as shown in Fig. 10. Bland–Altman analysis indicated strong agreement between the two methods in recovering CBFi, and both demonstrated comparable accuracy and robustness in rCBFi recovery. These results suggest that it is feasible to use a simpler model for rCBFi estimation, without requiring prior knowledge of $\mu_{a}$ and $\mu_{s}^{\prime }$. However, it is important to employ an appropriate optimization method, such as *fminsearchbnd*, and to avoid using *lsqcurvefit*, which showed inferior performance in this context.

As demonstrated in Fig. 6, CBFi sensitivity of the semi-infinite model increases with larger ρ, whereas the two-layer and three-layer models maintain CBFi sensitivity closer to the ground truth across all ρ. These results align with the findings shown in Figs. 2(a), 3(b), and 4(c): multi-layer models exhibit sufficient responsiveness to CBFi changes and can accurately recover absolute CBFi. However, as sensitivity fluctuates significantly at large ρ due to decreased SNR, the multi-layer models’ CBFi estimates also diverge considerably, potentially compromising reliability.

We further compared the three models’ abilities to eliminate the influence of extracerebral layers (SBFi and BBFi). As shown in Figs. 8 and 9, the two-layer model most effectively accounts for BFi variations in extracerebral layers among all models. The semi-infinite model consistently shows positive sensitivity to SBFi and BBFi variations, indicating that its recovered CBFi is strongly correlated with blood flow changes in extracerebral layers. Zhao et al.^44^ reported that semi-infinite model-derived rCBFi is unaffected by extracerebral layer changes only when CBFi and SBFi change by the same fractional amount, which is a highly restrictive condition. We believe that if a model exhibits non-zero sensitivity to changes in extracerebral blood flow, the recovered rCBFi is likely to be affected as well. Notably, the three-layer model appears more influenced by extracerebral BFi changes than the two-layer model [see Figs. 8(c) and 9(c)]. For example, although BBFi is relatively small, the three-layer model shows positive sensitivity to BBFi changes, resulting in increased recovered CBFi as BBFi increases. This effect likely arises because BBFi was assumed to be static during the fitting process, as is commonly done in multi-layer models;^46^ consequently, variations in BBFi are attributed to CBFi. Incorporating additional separations may enable BBFi to be treated as a fitting variable while preserving convergence, which we plan to investigate in future work. This may also be due to model bias when using a three-layer model to interpret a more complex anatomical structure, especially because MD fitting can drive the optimal solution away from the ground truth, which researchers in this field should pay attention to. Another possible reason is the small discrepancies we observed between MC simulations and the analytical solution of the CDE, as also reported by Zhao and Buckley.^57^ This represents a drawback of MD fitting that should be carefully considered.

Finally, we compared the time-to-result of different models using their corresponding fitting strategies to evaluate their suitability for CBFi monitoring. As listed in Table 4, the semi-infinite ETLR fitting and single-exponential ETLR fitting required significantly less time to process 500 data samples. In contrast, the two-layer and three-layer models were 25,000 and 92,367 times slower than the semi-infinite fitting, respectively. From a practical monitoring perspective, only the semi-infinite and single-exponential fittings are currently feasible for real-time applications. Encouragingly, deep learning (DL) methods have recently been introduced for DCS data processing and can greatly accelerate CBFi inference. However, most DCS-DL models have been developed based on the semi-infinite model, with only a few studies exploring multi-layer DCS models.^82^^,^^84^ Based on our results, we believe multi-layer DCS models hold significant promise for accurate CBFi monitoring but will likely require advanced data processing techniques to be practically feasible.

Although we have comprehensively compared the three DCS analytical models, there are several limitations in our study that could be addressed in future work. First, for ETLR fitting, we constrained the lag time to τ≤30  μs to simplify data analysis, whereas some previous studies have used criteria such as ≥1.2 or 1.25.^22^^,^^75^ As shown in Fig. 1, ETLR selects different portions of the curve at different ρ values, which may affect CBFi sensitivity; we did not perform a quantitative analysis of this influence. Second, as demonstrated in Sec. 3.1, we selected MD fitting for the three-layer model because it provides the most accurate estimates of both CBFi and SBFi. However, three-layer MD fitting tends to be sensitive to BFi changes in the extracerebral layers. To further evaluate the three-layer model’s ability to separate extracerebral confounders, a comparison using three-layer SD fitting should also be conducted. Moreover, in this study, we only considered a single pair of ρ for MD fitting, with one short separation fixed at 15 mm based on previous published works.^25^^,^^44^^,^^46^ We recognize that the optimal choice of separations is likely dependent on scalp and skull thicknesses. Although including additional separations could improve convergence and stability of the fitting, it also makes the computation substantially more demanding. Future work will focus on identifying optimal and practical separation strategies for MD fitting. Third, we generated our dataset using a four-layer slab model to represent the human head. Although this approach has been reported to introduce only small errors,^6^^,^^59^ future work should consider more complex and realistic head models to refine certain analyses. In addition, in our simulations, the thicknesses of the extracerebral layers were kept constant. However, variations in layer thickness can influence brain sensitivity at different ρ values. Therefore, future work should explore simulations incorporating varying extracerebral layer thicknesses to better understand these effects. Fourth, in adding noise to the simulated curves, we assumed a single photon count rate. Future studies should explore a broader range of noise levels to assess the robustness of different models under varied experimental conditions.

## 5 Conclusion

We systematically compared three CW-DCS models: the semi-infinite, two-layer, and three-layer models. We evaluated different fitting strategies, intrinsic CBFi sensitivity, accuracy of CBFi and rCBFi recovery, performance in separating extracerebral blood flow influence, sensitivity to errors in assumed parameters, and the feasibility of real-time CBFi monitoring. Our findings indicate that the semi-infinite model is suitable for robust rCBFi estimation at large ρ values, the two-layer model performs well for both CBFi and rCBFi estimation across all ρ (≥20  mm in this study), and the three-layer model is the most suitable for simultaneously extracting CBFi, SBFi, and rCBFi across all ρ. Among the three models, the two-layer model demonstrated the greatest robustness to BFi variations in extracerebral layers. Both the semi-infinite fitting and single-exponential fitting are suitable for real-time monitoring tasks. We believe this work offers a valuable reference for researchers aiming to accurately and appropriately apply DCS analytical models for CBFi quantification.

## Supplementary Material

10.1117/1.JBO.30.11.115002.s01

## Data Availability

The data underlying the results presented in this paper are not publicly available at this time but may be obtained from the authors upon reasonable request.

## References

[1] Carvajal K. Moreno-Sánchez R., “Heart metabolic disturbances in cardiovascular diseases,” Arch. Med. Res. 34(2), 89–99 (2003). 10.1016/S0188-4409(03)00004-3 12700003

[2] Durduran T. Yodh A. G., “Diffuse correlation spectroscopy for non-invasive, micro-vascular cerebral blood flow measurement,” NeuroImage 85, 51–63 (2014). NEIMEF 1053-8119 10.1016/j.neuroimage.2013.06.017 23770408 PMC3991554

[3] Boas D. A. Campbell L. E. Yodh A. G., “Scattering and imaging with diffusing temporal field correlations,” Phys. Rev. Lett. 75(9), 1855–1858 (1995). PRLTAO 0031-9007 10.1103/PhysRevLett.75.1855 10060408

[4] Gagnon L. et al., “Investigation of diffuse correlation spectroscopy in multi-layered media including the human head,” Opt. Express 16(20), 15514 (2008). OPEXFF 1094-4087 10.1364/OE.16.015514 18825190

[r5] Wang D. et al., “Fast blood flow monitoring in deep tissues with real-time software correlators,” Biomed. Opt. Express 7(3), 776 (2016). BOEICL 2156-7085 10.1364/BOE.7.000776 27231588 PMC4866455

[6] Zhao H. Buckley E. M., “Influence of oversimplifying the head anatomy on cerebral blood flow measurements with diffuse correlation spectroscopy,” Neurophotonics 10(1), 015010 (2023). 10.1117/1.NPh.10.1.015010 37006324 PMC10062384

[7] Busch D. R. et al., “Cerebral blood flow response to hypercapnia in children with obstructive sleep apnea syndrome,” Sleep 39(1), 209–216 (2016). SLEED6 0161-8105 10.5665/sleep.5350 26414896 PMC4678358

[r8] Nourhashemi M. et al., “Preictal neuronal and vascular activity precedes the onset of childhood absence seizure: direct current potential shifts and their correlation with hemodynamic activity,” Neurophotonics 10(2), 025005 (2023). 10.1117/1.NPh.10.2.025005 37114185 PMC10128878

[9] Lee S. Y. et al., “Quantifying the cerebral hemometabolic response to blood transfusion in pediatric sickle cell disease with diffuse optical spectroscopies,” Front. Neurol. 13, 869117 (2022). 10.3389/fneur.2022.869117 35847200 PMC9283827

[10] Durduran T. et al., “Transcranial optical monitoring of cerebrovascular hemodynamics in acute stroke patients,” Opt. Express 17(5), 3884 (2009). OPEXFF 1094-4087 10.1364/OE.17.003884 19259230 PMC2724658

[r11] Mesquita R. C. et al., “Diffuse optical characterization of an exercising patient group with peripheral artery disease,” J. Biomed. Opt. 18(5), 057007 (2013). JBOPFO 1083-3668 10.1117/1.JBO.18.5.057007 23708193 PMC3662991

[r12] Kaya K. et al., “Intraoperative cerebral hemodynamic monitoring during carotid endarterectomy via diffuse correlation spectroscopy and near-infrared spectroscopy,” Brain Sci. 12(8), 1025 (2022). 10.3390/brainsci12081025 36009088 PMC9405597

[13] Lafontant A. et al., “Comparison of optical measurements of critical closing pressure acquired before and during induced ventricular arrhythmia in adults,” Neurophotonics 9(3), 035004 (2022). 10.1117/1.NPh.9.3.035004 36039170 PMC9407009

[14] Udina C. et al., “Dual-task related frontal cerebral blood flow changes in older adults with mild cognitive impairment: a functional diffuse correlation spectroscopy study,” Front. Aging Neurosci. 14, 958656 (2022). 10.3389/fnagi.2022.958656 36605362 PMC9807627

[15] Cowdrick K. R. et al., “Agreement in cerebrovascular reactivity assessed with diffuse correlation spectroscopy across experimental paradigms improves with short separation regression,” Neurophotonics 10(2), 025002 (2023). 10.1117/1.NPh.10.2.025002 37034012 PMC10079775

[16] Dong L. et al., “Diffuse optical measurements of head and neck tumor hemodynamics for early prediction of chemoradiation therapy outcomes,” J. Biomed. Opt. 21(8), 085004 (2016). JBOPFO 1083-3668 10.1117/1.JBO.21.8.085004 27564315 PMC4999482

[17] Dong L. et al., “Noninvasive diffuse optical monitoring of head and neck tumor blood flow and oxygenation during radiation delivery,” Biomed. Opt. Express 3(2), 259 (2012). BOEICL 2156-7085 10.1364/BOE.3.000259 22312579 PMC3269843

[18] Yu G., “Diffuse correlation spectroscopy (DCS): a diagnostic tool for assessing tissue blood flow in vascular-related diseases and therapies,” Curr. Med. Imaging Rev. 8(3), 194–210 (2012). 10.2174/157340512803759875

[19] Boas D. A. Yodh A. G., “Spatially varying dynamical properties of turbid media probed with diffusing temporal light correlation,” J. Opt. Soc. Am. A 14(1), 192 (1997). JOAOD6 0740-3232 10.1364/JOSAA.14.000192

[20] Collins D. L. et al., “Design and construction of a realistic digital brain phantom,” IEEE Trans. Med. Imaging 17(3), 463–468 (1998). ITMID4 0278-0062 10.1109/42.712135 9735909

[21] Heiskala J. et al., “Modeling anisotropic light propagation in a realistic model of the human head,” Appl. Opt. 44(11), 2049–2057 (2005). APOPAI 0003-6935 10.1364/AO.44.002049 15835354

[22] Wu M. M. et al., “Improved accuracy of cerebral blood flow quantification in the presence of systemic physiology cross-talk using multi-layer Monte Carlo modeling,” Neurophotonics 8(1), 015001 (2021). 10.1117/1.NPh.8.1.015001 33437846 PMC7779997

[r23] Forti R. M. et al., “Optimizing a two-layer method for hybrid diffuse correlation spectroscopy and frequency-domain diffuse optical spectroscopy cerebral measurements in adults,” Neurophotonics 10(2), 025008 (2023). 10.1117/1.NPh.10.2.025008 37228905 PMC10204866

[r24] Selb J. et al., “Sensitivity of near-infrared spectroscopy and diffuse correlation spectroscopy to brain hemodynamics: simulations and experimental findings during hypercapnia,” Neurophotonics 1(1), 015005 (2014). 10.1117/1.NPh.1.1.015005 25453036 PMC4247161

[r25] Verdecchia K. et al., “Assessment of a multi-layered diffuse correlation spectroscopy method for monitoring cerebral blood flow in adults,” Biomed. Opt. Express 7(9), 3659 (2016). BOEICL 2156-7085 10.1364/BOE.7.003659 27699127 PMC5030039

[r26] Milej D. et al., “Direct assessment of extracerebral signal contamination on optical measurements of cerebral blood flow, oxygenation, and metabolism,” Neurophotonics 7(4), 045002 (2020). 10.1117/1.NPh.7.4.045002 33062801 PMC7540337

[r27] Dumont V. et al., “Somatosensory prediction in the premature neonate brain,” Dev. Cogn. Neurosci. 57, 101148 (2022). 10.1016/j.dcn.2022.101148 36027649 PMC9428805

[28] Wang Q. et al., “Quantification of blood flow index in diffuse correlation spectroscopy using a robust deep learning method,” J. Biomed. Opt. 29(1), 015004 (2024). JBOPFO 1083-3668 10.1117/1.JBO.29.1.015004 38283935 PMC10821781

[29] Durduran T. et al., “Diffuse optical measurement of blood flow, blood oxygenation, and metabolism in a human brain during sensorimotor cortex activation,” Opt. Lett. 29(15), 1766 (2004). OPLEDP 0146-9592 10.1364/OL.29.001766 15352363

[30] Wu M. M. et al., “Complete head cerebral sensitivity mapping for diffuse correlation spectroscopy using subject-specific magnetic resonance imaging models,” Biomed. Opt. Express 13(3), 1131 (2022). BOEICL 2156-7085 10.1364/BOE.449046 35414976 PMC8973189

[31] Farzam P. Durduran T., “Multidistance diffuse correlation spectroscopy for simultaneous estimation of blood flow index and optical properties,” J. Biomed. Opt. 20(5), 055001 (2015). JBOPFO 1083-3668 10.1117/1.JBO.20.5.055001 25938205

[32] Baker W. B. et al., “Pressure modulation algorithm to separate cerebral hemodynamic signals from extracerebral artifacts,” Neurophotonics 2(3), 035004 (2015). 10.1117/1.NPh.2.3.035004 26301255 PMC4524732

[33] Li J. et al., “Analytical models for time-domain diffuse correlation spectroscopy for multi-layer and heterogeneous turbid media,” Biomed. Opt. Express 8(12), 5518 (2017). BOEICL 2156-7085 10.1364/BOE.8.005518 29296485 PMC5745100

[r34] Sutin J. et al., “Time-domain diffuse correlation spectroscopy,” Optica 3(9), 1006 (2016). 10.1364/OPTICA.3.001006 28008417 PMC5166986

[35] Samaei S. et al., “Time-domain diffuse correlation spectroscopy (TD-DCS) for noninvasive, depth-dependent blood flow quantification in human tissue in vivo,” Sci. Rep. 11(1), 1817 (2021). SRCEC3 2045-2322 10.1038/s41598-021-81448-5 33469124 PMC7815740

[36] Kholiqov O. et al., “Time-of-flight resolved light field fluctuations reveal deep human tissue physiology,” Nat. Commun. 11(1), 391 (2020). NCAOBW 2041-1723 10.1038/s41467-019-14228-5 31959896 PMC6971031

[37] Robinson M. B. et al., “Portable, high speed blood flow measurements enabled by long wavelength, interferometric diffuse correlation spectroscopy (LW-iDCS),” Sci. Rep. 13(1), 8803 (2023). SRCEC3 2045-2322 10.1038/s41598-023-36074-8 37258644 PMC10232495

[38] Carp S. A. et al., “Diffuse correlation spectroscopy measurements of blood flow using 1064 nm light,” J. Biomed. Opt. 25(9), 097003 (2020). JBOPFO 1083-3668 10.1117/1.JBO.25.9.097003 32996299 PMC7522668

[39] Sie E. J. et al., “High-sensitivity multispeckle diffuse correlation spectroscopy,” Neurophotonics 7(3), 035010 (2020). 10.1117/1.NPh.7.3.035010 32995362 PMC7519351

[40] Wayne M. A. et al., “Massively parallel, real-time multispeckle diffuse correlation spectroscopy using a 500 × 500 SPAD camera,” Biomed. Opt. Express 14(2), 703 (2023). BOEICL 2156-7085 10.1364/BOE.473992 36874503 PMC9979680

[41] Li J. et al., “Noninvasive detection of functional brain activity with near-infrared diffusing-wave spectroscopy,” J. Biomed. Opt. 10(4), 044002 (2005). JBOPFO 1083-3668 10.1117/1.2007987 16178636

[r42] Baker W. B. et al., “Modified Beer-Lambert law for blood flow,” Biomed. Opt. Express 5(11), 4053 (2014). BOEICL 2156-7085 10.1364/BOE.5.004053 25426330 PMC4242038

[43] Kholiqov O. et al., “Scanning interferometric near-infrared spectroscopy,” Opt. Lett. 47(1), 110 (2022). OPLEDP 0146-9592 10.1364/OL.443533 34951892 PMC9281567

[44] Zhao H. Sathialingam E. Buckley E. M., “Accuracy of diffuse correlation spectroscopy measurements of cerebral blood flow when using a three-layer analytical model,” Biomed. Opt. Express 12(11), 7149 (2021). BOEICL 2156-7085 10.1364/BOE.438303 34858706 PMC8606134

[45] Carp S. A. et al., “Recovery of brain blood flow changes from diffuse correlation spectroscopy data using a layered Monte Carlo forward model,” in Biomedical Optics 2014, pp. BM3A.20, OSA, Miami, Florida (2014).

[46] Zhao H. et al., “Comparison of diffuse correlation spectroscopy analytical models for measuring cerebral blood flow in adults,” J. Biomed. Opt. 28(12), 126005 (2023). JBOPFO 1083-3668 10.1117/1.JBO.28.12.126005 38107767 PMC10723621

[47] Haskell R. C. et al., “Boundary conditions for the diffusion equation in radiative transfer,” J. Opt. Soc. Am. A 11(10), 2727 (1994). JOAOD6 0740-3232 10.1364/JOSAA.11.002727 7931757

[48] Boas D. A., Diffuse Photon Probes of Structural and Dynamical Properties of Turbid Media: Theory and Biomedical Applications, University of Pennsylvania (1996).

[49] Case K. M. Zweifel P. F. Pomraning G. C., “Linear transport theory,” Phys. Today 21(10), 72–73 (1968). PHTOAD 0031-9228 10.1063/1.3034554

[50] Ackerson B. J. et al., “Correlation transfer—application of radiative transfer solution methods to photon correlation problems,” J. Thermophys. Heat Transf. 6(4), 577–588 (1992). JTHTEO 0887-8722 10.2514/3.11537

[51] Irwin D. et al., “Influences of tissue absorption and scattering on diffuse correlation spectroscopy blood flow measurements,” Biomed. Opt. Express 2(7), 1969 (2011). BOEICL 2156-7085 10.1364/BOE.2.001969 21750773 PMC3130582

[52] Durduran T., “Noninvasive measurements of tissue hemodynamics with hybrid diffuse optical methods,” Med. Phys. 31(7), 2178–2178 (2004). MPHYA6 0094-2405 10.1118/1.1763412

[53] Boas D. A. et al., “Establishing the diffuse correlation spectroscopy signal relationship with blood flow,” Neurophotonics 3(3), 031412 (2016). 10.1117/1.NPh.3.3.031412 27335889 PMC4904065

[54] Sathialingam E. et al., “Small separation diffuse correlation spectroscopy for measurement of cerebral blood flow in rodents,” Biomed. Opt. Express 9(11), 5719–5734 (2018). BOEICL 2156-7085 10.1364/BOE.9.005719 30460158 PMC6238900

[55] Pine D. J. et al., “Diffusing wave spectroscopy,” Phys. Rev. Lett. 60(12), 1134–1137 (1988). PRLTAO 0031-9007 10.1103/PhysRevLett.60.1134 10037950

[56] Fang Q. Boas D. A., “Monte Carlo simulation of photon migration in 3D turbid media accelerated by graphics processing units,” Opt. Express 17(22), 20178 (2009). OPEXFF 1094-4087 10.1364/OE.17.020178 19997242 PMC2863034

[57] Zhao H. Buckley E. M., “Influence of source–detector separation on diffuse correlation spectroscopy measurements of cerebral blood flow with a multilayered analytical model,” Neurophotonics 9(3), 035002 (2022). 10.1117/1.NPh.9.3.035002 35874143 PMC9299346

[58] Jaillon F. et al., “Diffusing-wave spectroscopy from head-like tissue phantoms: influence of a non-scattering layer,” Opt. Express 14(22), 10181 (2006). OPEXFF 1094-4087 10.1364/OE.14.010181 19529414

[59] Custo A. et al., “Effective scattering coefficient of the cerebral spinal fluid in adult head models for diffuse optical imaging,” Appl. Opt. 45(19), 4747–4755 (2006). APOPAI 0003-6935 10.1364/AO.45.004747 16799690

[60] Okada E. et al., “Theoretical and experimental investigation of near-infrared light propagation in a model of the adult head,” Appl. Opt. 36(1), 21 (1997). APOPAI 0003-6935 10.1364/AO.36.000021 18250644

[61] Yaroslavsky A. N. et al., “Optical properties of selected native and coagulated human brain tissues in vitro in the visible and near infrared spectral range,” Phys. Med. Biol. 47(12), 2059 (2002). PHMBA7 0031-9155 10.1088/0031-9155/47/12/305 12118601

[r62] Strangman G. Franceschini M. A. Boas D. A., “Factors affecting the accuracy of near-infrared spectroscopy concentration calculations for focal changes in oxygenation parameters,” NeuroImage 18(4), 865–879 (2003). NEIMEF 1053-8119 10.1016/S1053-8119(03)00021-1 12725763

[r63] Mazumder D. et al., “Optimization of time domain diffuse correlation spectroscopy parameters for measuring brain blood flow,” Neurophotonics 8(3), 035005 (2021). 10.1117/1.NPh.8.3.035005 34395719 PMC8358828

[64] Okada E. Delpy D. T., “Near-infrared light propagation in an adult head model I: modeling of low-level scattering in the cerebrospinal fluid layer,” Appl. Opt. 42(16), 2906 (2003). APOPAI 0003-6935 10.1364/AO.42.002906 12790439

[65] Boas D. A. et al., “Three dimensional Monte Carlo code for photon migration through complex heterogeneous media including the adult human head,” Opt. Express 10(3), 159–170 (2002). OPEXFF 1094-4087 10.1364/OE.10.000159 19424345

[66] Selb J. et al., “Improved sensitivity to cerebral hemodynamics during brain activation with a time-gated optical system: analytical model and experimental validation,” J. Biomed. Opt. 10(1), 011013 (2005). JBOPFO 1083-3668 10.1117/1.1852553 15847579

[67] Barnett A. H. et al., “Robust inference of baseline optical properties of the human head with three-dimensional segmentation from magnetic resonance imaging,” Appl. Opt. 42(16), 3095 (2003). APOPAI 0003-6935 10.1364/AO.42.003095 12790461

[68] Ohmae E. et al., “Cerebral hemodynamics evaluation by near-infrared time-resolved spectroscopy: correlation with simultaneous positron emission tomography measurements,” NeuroImage 29(3), 697–705 (2006). NEIMEF 1053-8119 10.1016/j.neuroimage.2005.08.008 16165372

[69] Rangel de Lázaro G. et al., “The endocranial vascular system: tracing vessels,” in Digital Endocasts: from Skulls to Brains, Bruner E. Ogihara N. and Tanabe H. C., Eds., pp. 71–91, Springer Japan, Tokyo (2018).

[70] Zhou C. et al., “Diffuse optical correlation tomography of cerebral blood flow during cortical spreading depression in rat brain,” Opt. Express 14(3), 1125 (2006). OPEXFF 1094-4087 10.1364/OE.14.001125 19503435

[71] American National Standards Institute and Laser Institute of America, American National Standard for safe use of lasers: approved March 16, 2007, The Laser Institute of America, Orlando, Florida (2007).

[72] Durduran T. et al., “Diffuse optics for tissue monitoring and tomography,” Rep. Prog. Phys. 73(7), 076701 (2010). RPPHAG 0034-4885 10.1088/0034-4885/73/7/076701 26120204 PMC4482362

[73] Zhou C., “In-vivo optical imaging and spectroscopy of cerebral hemodynamics,” PhD in ProQuest Dissertations and Theses, University of Pennsylvania (2007).

[74] Dong L. et al., “Simultaneously extracting multiple parameters via fitting one single autocorrelation function curve in diffuse correlation spectroscopy,” IEEE Trans. Biomed. Eng. 60(2), 361–368 (2013). IEBEAX 0018-9294 10.1109/TBME.2012.2226885 23193446 PMC4337031

[75] Buckley E. M. et al., “Validation of diffuse correlation spectroscopic measurement of cerebral blood flow using phase-encoded velocity mapping magnetic resonance imaging,” J. Biomed. Opt. 17(3), 037007 (2012). JBOPFO 1083-3668 10.1117/1.JBO.17.3.037007 22502579 PMC3380925

[76] Gorman A. et al., “ATLAS: a large array, on-chip compute SPAD camera for multispeckle diffuse correlation spectroscopy,” Biomed. Opt. Express 15(11), 6499–6515 (2024). BOEICL 2156-7085 10.1364/BOE.531416 39553854 PMC11563329

[77] Poon C.-S. Long F. Sunar U., “Deep learning model for ultrafast quantification of blood flow in diffuse correlation spectroscopy,” Biomed. Opt. Express 11(10), 5557 (2020). BOEICL 2156-7085 10.1364/BOE.402508 33149970 PMC7587273

[78] Cheng X. et al., “Development of a Monte Carlo-wave model to simulate time domain diffuse correlation spectroscopy measurements from first principles,” J. Biomed. Opt. 27(8), 083009 (2022). JBOPFO 1083-3668 10.1117/1.JBO.27.8.083009 35199501 PMC8866418

[79] Fercher A. F. Briers J. D., “Flow visualization by means of single-exposure speckle photography,” Opt. Commun. 37(5), 326–330 (1981). OPCOB8 0030-4018 10.1016/0030-4018(81)90428-4

[80] Buckley E. M. et al., “Diffuse correlation spectroscopy for measurement of cerebral blood flow: future prospects,” Neurophotonics 1(1), 011009 (2014). 10.1117/1.NPh.1.1.011009 25593978 PMC4292799

[81] Tachtsidis I. Scholkmann F., “False positives and false negatives in functional near-infrared spectroscopy: issues, challenges, and the way forward,” Neurophotonics 3(3), 031405 (2016). 10.1117/1.NPh.3.3.031405 27054143 PMC4791590

[82] Pan M. et al., “Cerebral blood flow monitoring using a deep learning implementation of the two-layer diffuse correlation spectroscopy analytical model with a 512 × 512 SPAD array,” Neurophotonics 12(3), 035008 (2025). 10.1117/1.NPh.12.3.035008 40831579 PMC12360787

[83] Wang Q. et al., “A comprehensive overview of diffuse correlation spectroscopy: theoretical framework, recent advances in hardware, analysis, and applications,” NeuroImage 298, 120793 (2024). NEIMEF 1053-8119 10.1016/j.neuroimage.2024.120793 39153520

[84] Zang Z. et al., “Fast blood flow index reconstruction of diffuse correlation spectroscopy using a back-propagation-free data-driven algorithm,” Biomed. Opt. Express 16(3), 1254–1269 (2025). BOEICL 2156-7085 10.1364/BOE.549363 40109530 PMC11919341

