# Supplementary material for: Comparison of diffuse correlation spectroscopy analytical models for cerebral blood flow measurements
Source: J Biomed Opt. 2025 Nov 12;30(11):115002. doi: 10.1117/1.JBO.30.11.115002 (PMC12617376; doi:10.1117/1.JBO.30.11.115002)
Supplement: Supplementary file 1 [file JBO_030_115002_SD001.docx]

Comparison of diffuse correlation spectroscopy analytical models for cerebral blood flow measurements

Mingliang Pan^*^, Quan Wang, Yuanzhe Zhang, and David Day-Uei Li^*^

*Mingliang Pan, E-mail: [mingliang.pan@strath.ac.uk](mailto:mingliang.pan@strath.ac.uk); David Day-Uei Li, E-mail: [david.li@strath.ac.uk](mailto:david.li@strath.ac.uk)

**Supplementary Content:**

1. DCS analytical models’ derivation

*Semi-infinite model*

For a semi-infinite homogeneous medium, the correlation diffusion equation can be expressed as:

$$\begin{aligned} \left( \frac{D}{\upsilon}\nabla^{2}-\mu_{a}-\frac{1}{3}\mu_{s}^{'}k_{0}^{2}\alpha\left\langle\Delta r^{2}\left( \tau\right) \right\rangle\right)G_{1}\left( \mathbf{r},\tau\right)=-S\left( \mathbf{r} \right),\#\left( seq equation 1 \right) \end{aligned}$$

The analytical solution to Eq. (1) under the assumption of extrapolated boundary conditions, is given by:

$$\begin{aligned} G_{1}\left( \rho,\tau\right)=\frac{3\mu_{s}^{'}}{4\pi}\left[ \frac{e^{\left( -Kr_{1} \right)}}{r_{1}}-\frac{e^{\left( -Kr_{2} \right)}}{r_{2}} \right],\#\left( seq equation 2 \right) \end{aligned}$$

where $D_{r}=\upsilon/\left( 3\mu_{s}^{'} \right)$ is the photon diffusion coefficient, $\upsilon$ is the speed of light in the medium, $\tau$ is the delay time, $\rho$ is the source-detector separation, $K^{2}=3\mu_{a}\mu_{s}^{'}+{\mu_{s}^{'}}^{2}k_{0}^{2}\alpha\left\langle\Delta r^{2}\left( \tau\right) \right\rangle$, $k_{0}$ is the wavenumber of light in the medium, $\mu_{a}$is the absorption coefficient, $\mu_{s}^{'}$ is the reduced scattering coefficient, $r_{1}=\sqrt{\rho^{2}+z_{0}^{2}}$, $r_{2}=\sqrt{\rho^{2}+\left( z_{0}+2z_{b} \right)^{2}}$, $z_{0}=1/\left( \mu_{a}+\mu_{s}^{'} \right)$, $z_{b}=2\left( 1+R_{\mathrm{eff}} \right)/\left( 3\mu_{s}^{'}\left( 1-R_{\mathrm{eff}} \right) \right)$ with $R_{\mathrm{eff}}=-1.44n^{-2}+0.71n^{-1}+0.668+0.064n$ being the effective reflection coefficient, defined by the ratio of the refraction indices inside and outside the medium (e.g., $n=n_{0}/n_{air}$, $n_{0}$ is the medium refractive index, $n_{air}$ is the air refraction index).

*Two-layer model*

For the two-layer model, we treat the tissue geometry as two slabs. The upper layer accounts for superficial tissues, and the under one represents deeper tissue. For example, when using the two-layer model to extract CBFi, we simplify human head structure as one layer to represent extracerebral tissue (scalp, skull), and another semi-infinite part to represent cerebral tissue (cerebrospinal fluid, grey matter, white matter, etc.). Following the analytical derivation process developed by Gagnon *et al*. (Ref. 4 in the main text), we assume an isotropic source incident at depth $z_{0}=1/\left( \mu_{a,1}+\mu_{s,1}^{'} \right)$, and scatters in each layer present independent Brownian diffusion motion. Then the CDE will be

$$\left[ D_{1}\nabla^{2}-\mu_{a,1}-2\mu_{s,1}^{'}k_{0}^{2}D_{B,1}\tau\right]G_{1}^{1}\left( x,y,z,\tau\right)=-\delta\left( x,y,z-z_{0} \right) 0\leq z\leq l,$$

$$\begin{aligned} \left[ D_{2}\nabla^{2}-\mu_{a,2}-2\mu_{s,2}^{'}k_{0}^{2}D_{B,2}\tau\right]G_{1}^{2}\left( x,y,z,\tau\right)=0 l\leq z,\#\left( 3 \right) \end{aligned}$$

where $j=1,2$ refers to the layer indices, $D_{j}$, $\mu_{a,j}$, $\mu_{s,j}^{'}$, and $D_{B,j}$ are the diffusion coefficient, absorption coefficient, reduced scattering coefficient, and Brownian diffusion coefficient in Layer $j$, respectively, $l$ is the thickness of Layer 1. The Fourier domain solution to Eq. (3) at the surface of Layer 1 is

$$\begin{aligned} \tilde{G}_{1}^{1}\left( s,z,\tau\right)=\frac{\sinh\left[ \alpha_{1}\left( z_{b}+z_{0} \right) \right]}{D_{1}\alpha_{1}}\times\frac{D_{1}\alpha_{1}\cosh\left[ \alpha_{1}\left( l-z \right) \right]+D_{2}\alpha_{2}\sinh\left[ \alpha_{1}\left( l-z \right) \right]}{D_{1}\alpha_{1}\cosh\left[ \alpha_{1}\left( l+z_{b} \right) \right]+D_{2}\alpha_{2}\sinh\left[ \alpha_{1}\left( l+z_{b} \right) \right]} \\ -\frac{\sinh\left[ \alpha_{1}\left( z_{0}-z \right) \right]}{D_{1}\alpha_{1}},\#\left( 4 \right) \end{aligned}$$

where $\alpha_{j}^{2}=\left( D_{j}s^{2}+\mu_{a,j}+2v\mu_{s,j}^{'}k_{0}^{2}D_{B,j} \right)/D_{j}$, $v$ is the light speed, $z_{b}=2D_{1}\left( 1+R_{\mathrm{eff}} \right)/\left( 1-R_{\mathrm{eff}} \right)$. The Fourier inversion of Eq. (4) is

$$\begin{aligned} G_{1}^{1}\left( \rho,z,\tau\right)=\frac{1}{2\pi}\int_{0}^{\infty} \tilde{G}_{1}^{1}\left( s,z,\tau\right)sJ_{0}\left( s\rho\right)ds,\#\left( 5 \right) \end{aligned}$$

where $J_{0}$ is the zeroth order Bessel function of the first kind.

*Three-layer model*

Similar to the two-layer model, we treat the tissue geometry as three slabs. For the human head, Layer 1 refers to scalp with a thickness $l_{1}$, Layer 2 refers to skull with a thickness $l_{2}$, and Layer 3 represents brain with a semi-infinite geometry type, i.e. $l_{3}\to\infty$. Under the same assumptions, the CDE using three-layer model will be

$$\begin{aligned} \left[ \nabla^{2}-3\mu_{a,j}\mu_{s,j}^{'}+6k_{0}^{2}{\mu_{s,j}^{'}}^{2}D_{B,j}\tau\right]G_{1}\left( \mathbf{r},\tau\right)=-s_{0}\delta\left( \mathbf{r-}\mathbf{r}^{\mathbf{'}} \right),\#\left( 6 \right) \end{aligned}$$

We solve Eq. (3) in Fourier domain, and we can obtain the Fourier domain electric field temporal autocorrelation function as

$$\begin{aligned} \tilde{G}_{1}^{0}\left( \mathbf{q},z=0,\tau\right)=\frac{\mathrm{Numerator}}{\mathrm{Denominator}},\#\left( 7 \right) \end{aligned}$$

$$\mathrm{Numerator}=s_{0}z_{0}\left\{ \beta_{1}D_{1}\cosh\left[ \beta_{1}\left( l_{1}-z_{b} \right) \right]\left[ \beta_{2}D_{2}\cosh\left( \beta_{2}l_{2} \right)+\beta_{3}D_{3}\sinh\left( \beta_{2}l_{2} \right) \right] \right\}$$

$$\begin{aligned} &+s_{0}z_{0}\left\{ \beta_{2}D_{2}\left[ \beta_{3}D_{3}\cosh\left( \beta_{2}l_{2} \right)+\beta_{2}D_{2}\sinh\left( \beta_{2}l_{2} \right) \right]\sinh\left[ \beta_{1}\left( l_{1}-z_{b} \right) \right] \right\},\#\left( 8 \right) \end{aligned}$$

$$\begin{aligned} \mathrm{Denominator}=&\beta_{2}D_{2}\cosh\left( \beta_{2}l_{2} \right)\left[ \beta_{1}\left( D_{1}+\beta_{3}D_{3}z_{0} \right)\cosh\left( \beta_{1}l_{1} \right) \right.\# \\ &+\left( \beta_{3}D_{3}+\beta_{1}^{2}D_{1}z_{0} \right)\sinh\left( \beta_{1}l_{1} \right)]\# \\ &+{[\beta}_{1}\left( \beta_{3}D_{1}D_{3}+\beta_{2}^{2}D_{2}^{2}z_{0} \right)\cosh\left( \beta_{1}l_{1} \right)\# \\ &+\left( \beta_{2}^{2}D_{2}^{2}+\beta_{1}^{2}\beta_{3}D_{1}D_{3}z_{0} \right)\sinh\left( \beta_{1}l_{1} \right)]\times\sinh\left( \beta_{2}l_{2} \right),\#\left( 9 \right) \end{aligned}$$

where $\beta_{j}^{2}\left( \mathbf{q},\tau\right)=3\mu_{a,j}\mu_{s,j}^{'}+6k_{0}^{2}{\mu_{s,j}^{'}}^{2}D_{B,j}\tau+\mathbf{q}^{2}$, $j=1, 2, 3$ refers to the layer indices, $D_{j}=\upsilon/\left( 3\mu_{s,j}^{'} \right)$, $\mu_{a,j}$, $\mu_{s,j}^{'}$, $l_{j}$ and $D_{B,j}$ are the diffusion coefficient, absorption coefficient, reduced scattering coefficient, layer thickness, and Brownian diffusion coefficient in Layer $j$, respectively, $\upsilon$ is the light speed. $z_{0}=1/\left( \mu_{a,1}+\mu_{s,1}^{'} \right)$, $z_{b}=2D_{1}\left( 1+R_{\mathrm{eff}} \right)/\left( 1-R_{\mathrm{eff}} \right)$

The measured field autocorrelation function at position $\mathbf{r}=\left\{ \boldsymbol{\rho},z=0 \right\}$ on the surface of the first layer will be (the inverse Fourier transform)

$$\begin{aligned} G_{1}^{0}\left( \mathbf{r},\tau\right)=&\frac{1}{\left( 2\pi\right)^{2}}\int d^{2}\mathbf{q}\tilde{G}_{1}^{0}\left( \mathbf{q},z=0,\tau\right)e^{-i\mathbf{q}\cdot\boldsymbol{\rho}} \\ =&\frac{1}{2\pi}\int d\mathbf{q}\tilde{G}_{1}^{0}\left( \mathbf{q},z=0,\tau\right)qJ_{0}\left( \rho q \right),\#\left( 10 \right) \end{aligned}$$

where $J_{0}$ denotes the zeroth order Bessel function of the first kind.

*2. SNR analysis of the synthesized experimental data*

We validated our noise model using experimental results obtained from measurements on a milk phantom. The milk (1.8% fat, semi-skimmed, purchased from ALDI grocery) was diluted with water at a ratio of 1:3. We developed a traditional DCS system consisting of a multimode fiber (600 *µm* core diameter, NA = 0.22, Thorlabs M160L01) delivering a 30 mW, 785 nm laser (DL785-100-S, CrystaLaser) to the sample. Scattered light was collected using a single-mode fiber (785HP, THORLABS) connected to a single-photon avalanche diode (SPAD; ID101-SMF20, Swiss Quantum). Photon arrival times were recorded and processed by a time-tagger module (SPC-QC-104, Becker & Hickl GmbH) to compute the intensity autocorrelation function, $g_{2}$. We then applied the semi-infinite analytical model to fit the experimental data. Fig. S1 shows both the raw $g_{2}$ curves and the corresponding fitted (analytical) curves.

To validate the noise model, we conducted experiments at two $\rho$ (20, 30 mm). The observed photon count rates at 20 mm and 30 mm were 121.1 kcps and 29.5 kcps, respectively. We set the integration time to 1 second. For each $\rho$, we collected 100 $g_{2}$ curves to calculate the standard deviation at each time lag $\tau$. The observed step-like pattern in the results is due to the multi-tau configuration of the hardware correlator, where the bin width increases stepwise (6.145 ns for the first 16-channel and is tripled every 16-channel thereafter). As stated in the main text, as a validation, the parameters used in our noise model were all derived from experimental settings and observations.

Fig. S2(a) illustrates the noise level (standard deviation, $\sigma$, at each$\tau$) at $\rho$ = 20 mm and 30 mm, validating our theoretical noise model against experimental measurements. Fig. S2(b) presents the SNR computed from both the theoretical noise model and the experimental analysis, using Eq. (6) from the main text. Both the noise and SNR analyses demonstrate excellent agreement between the theoretical noise model and experimental results as reported in previous studies, confirming the reliability of the noise-adding method employed in this study.


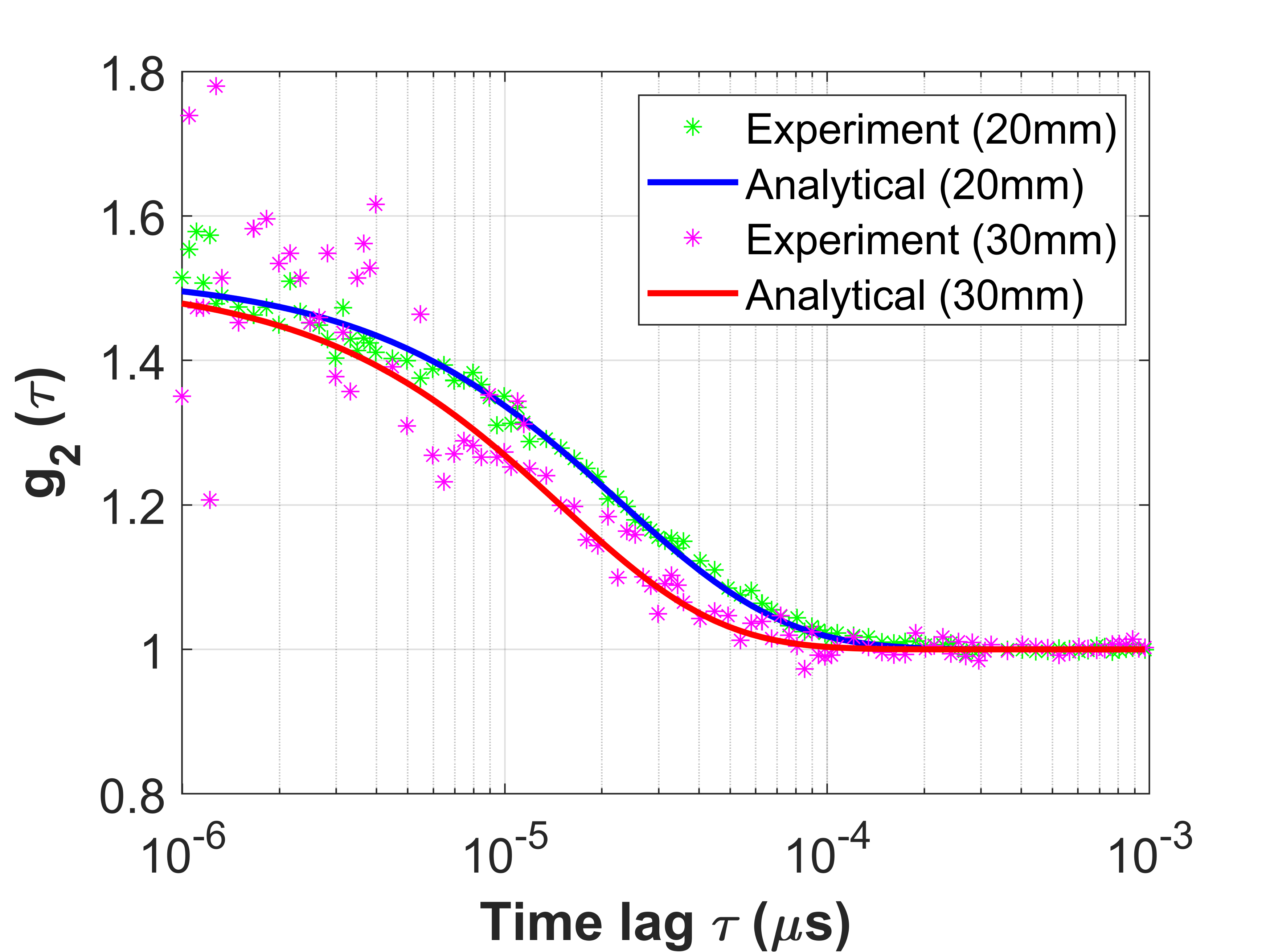


**Fig. S1** Noisy $g_{2}$ curves (scatter plots) alongside the fitted $g_{2}$ curves from the semi-infinite analytical model (solid lines) at $\rho$ = 20 mm and 30 mm.


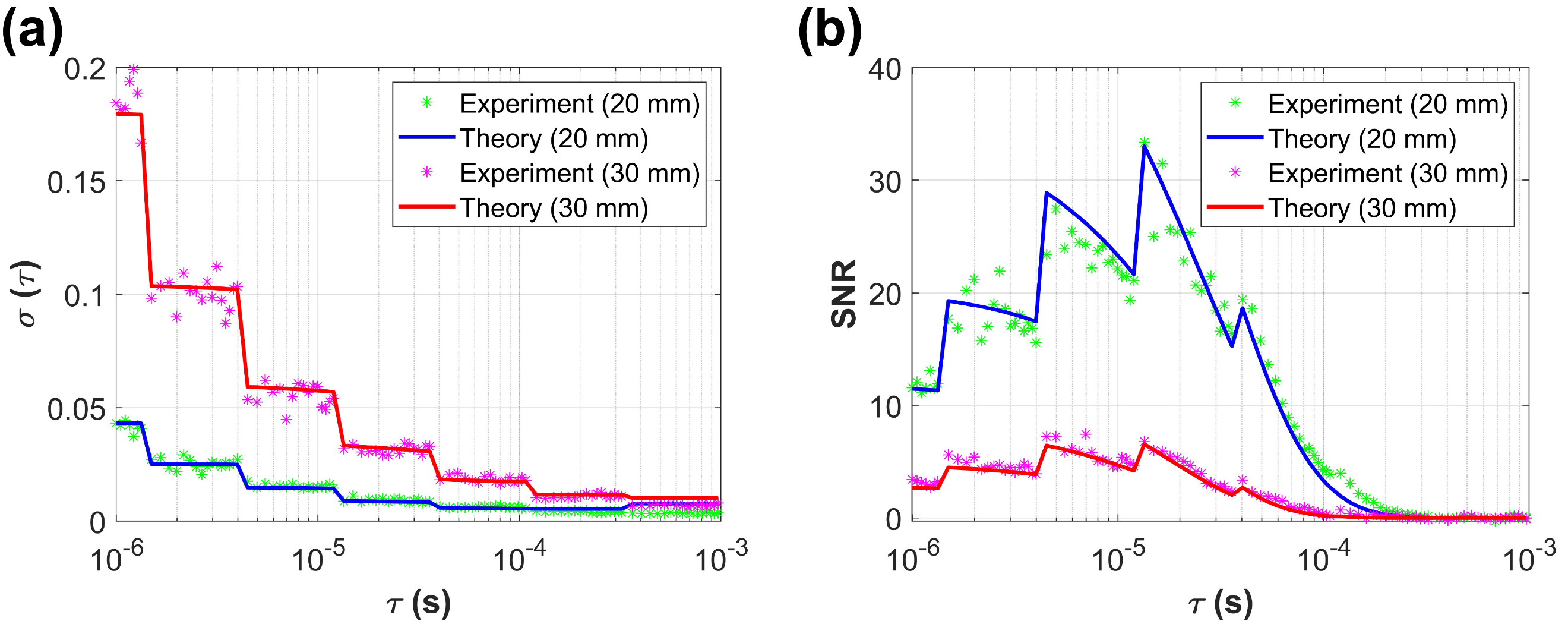


**Fig. S2** Validation of the noise model using the milk phantom experiment. (a) Standard deviation ($\sigma$) as a function of $\tau$ from the noise model, compared against experimental results at $\rho$ = 20 mm and 30 mm. (b) SNR calculated from the noise model, validated against experimental results. For the experimental analysis, each measurement was repeated 100 times to perform statistical analysis.

3. Two-layer MD fitting for rCBFi recovery

We performed two-layer MD fitting to estimate rCBFi. Compared to two-layer SD fitting, the two-layer MD fitting achieves better convergence at larger $\rho$ values (35 mm). However, the two-layer MD fitting tends to overestimate rCBFi across all $\rho$, suggesting that its CBFi sensitivity is higher than that of the SD fitting method, which may lead to inaccuracies in estimating CBFi changes.

*
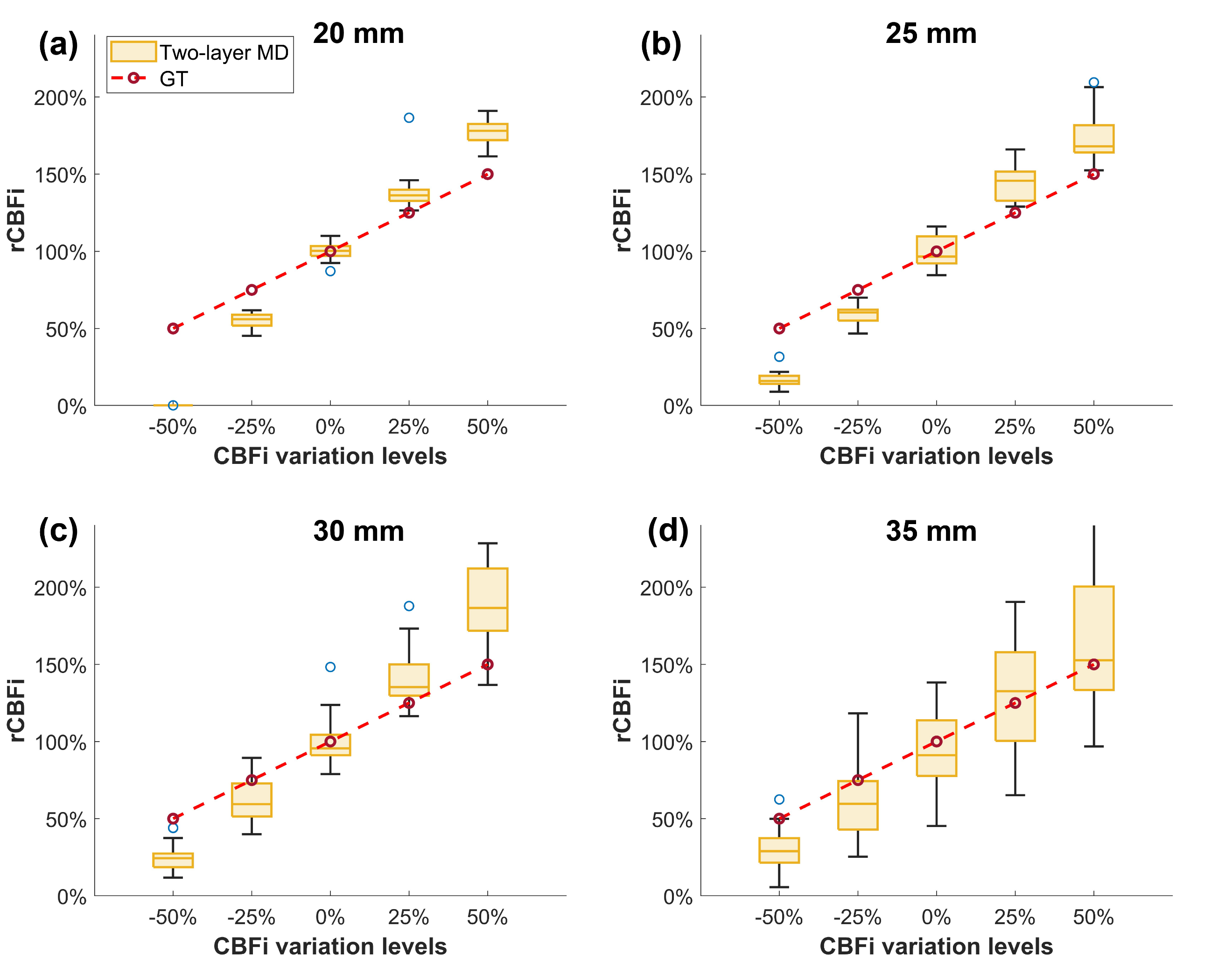
*

**Fig. S3** Two-layer MD fitting recovered rCBFi at different $\rho$ (20, 25, 30, 35 mm). The simulated CBFi perturbation has four levels, ±50% and ±25% compare to the baseline (6×10^-6^ mm^2^/s).

4. Comparison between fminsearchbnd and lsqcurvefit for single-exponential fitting

We compared rCBFi recovered by single-exponential fitting using the *fminsearchbnd* and *lsqcurvefit* functions in MATLAB. As shown in Fig. S4, the rCBFi estimated with *lsqcurvefit* exhibits greater fluctuations and more pronounced underestimation compared to results obtained using *fminsearchbnd*.


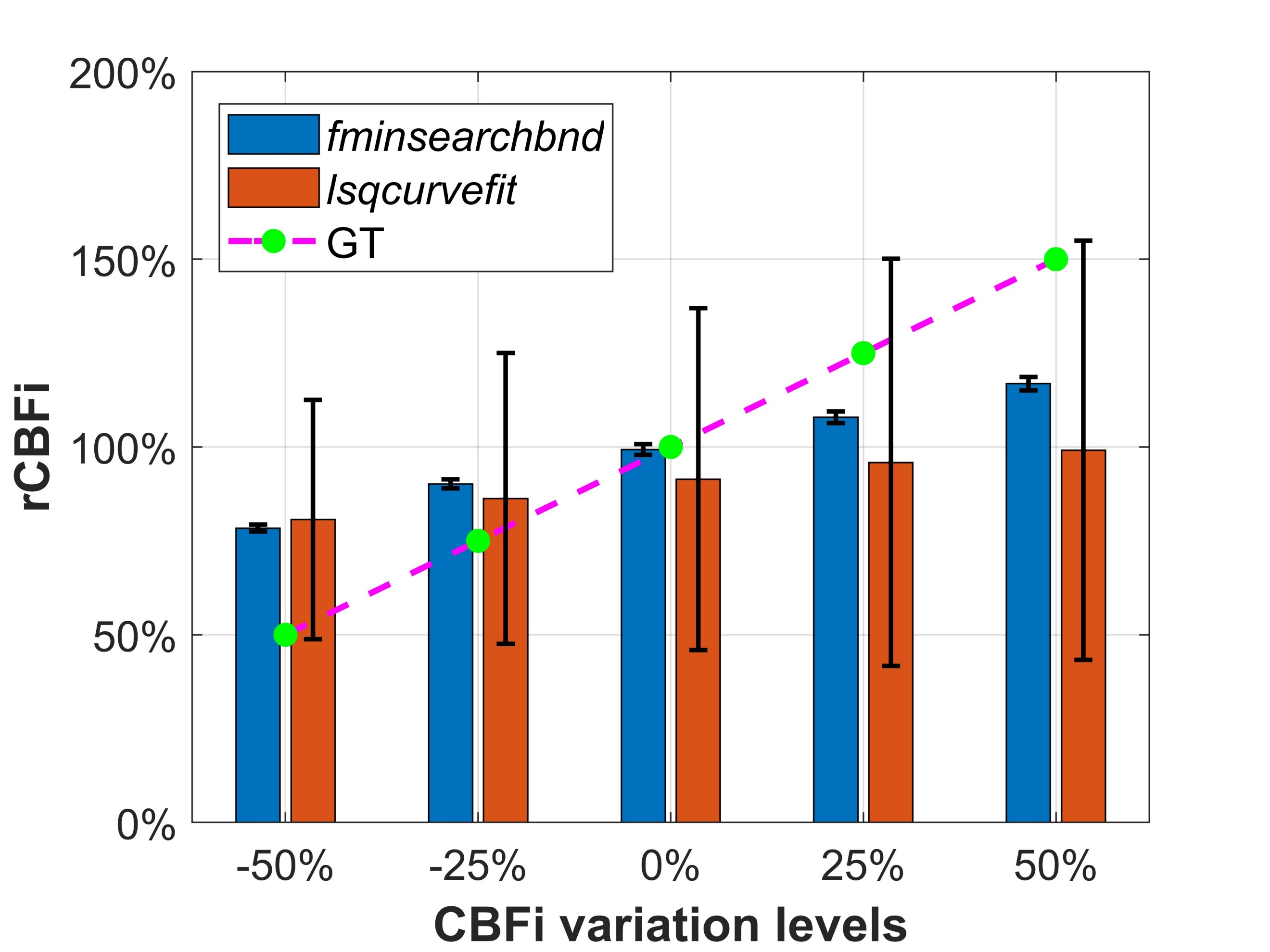


**Fig. S4** Single-exponential fitting recovered rCBFi using *fminsearchbnd* (blue) and *lsqcurvefit* (orange) functions. The bar represents the median of the recovered rCBFi, the error bar represents the 25th and 75th percentile range of the recovered rCBFi.
